# FOXA2 Suppression by TRIM36 Exerts Anti‐Tumor Role in Colorectal Cancer Via Inducing NRF2/GPX4‐Regulated Ferroptosis

**DOI:** 10.1002/advs.202304521

**Published:** 2023-10-24

**Authors:** Xin Liu, Chunli Yan, Chunxiao Chang, Fansong Meng, Wenjie Shen, Song Wang, Yi Zhang

**Affiliations:** ^1^ Department of Gastrointestinal Surgery Shandong Cancer Hospital and Institute Shandong First Medical University & Shandong Academy of Medical Sciences Jinan 250117 China; ^2^ Department of Breast Internal Medicine Shandong Cancer Hospital and Institute Shandong First Medical University & Shandong Academy of Medical Sciences Jinan 250117 China; ^3^ Ward 2 of Gastroenterology Shandong Cancer Hospital and Institute Shandong First Medical University & Shandong Academy of Medical Sciences Jinan 250117 China; ^4^ Department of Medical Management Shandong Cancer Hospital and Institute Shandong First Medical University & Shandong Academy of Medical Sciences Jinan 250117 China; ^5^ Clinical Trial Research Center Shandong Cancer Hospital and Institute Shandong First Medical University & Shandong Academy of Medical Sciences Jinan 250117 China

**Keywords:** colorectal cancer (CRC), FOXA2, ferroptosis, Nrf2/GPX4, TRIM36

## Abstract

The forkhead box transcription factor A2 (FOXA2) is a transcription factor and plays a key role in embryonic development, metabolism homeostasis and tumor cell proliferation; however, its regulatory potential in CRC is not fully understood. Here, it is found that FOXA2 expression is markedly up‐regulated in tumor samples of CRC patients as compared with the normal tissues, which is closely associated with the worse survival in patients with CRC. Notably, a positive correlation between FOXA2 and nuclear factor erythroid 2‐related factor 2 (Nrf2)/glutathione peroxidase 4 (GPX4) gene expression is observed in CRC patients. Mechanistically, FOXA2 depletion weakens the activation of Nrf2 pathway and decreases GPX4 level in CRC cells, thereby leading to ferroptosis, which is further supported by bioinformatic analysis. More intriguingly, the E3 ubiquitin ligase tripartite motif containing 36 (TRIM36) is identified as a key suppressor of FOXA2, and it is observed that TRIM36 can directly interact with FOXA2 and induce its K48‐linked polyubiquitination, resulting in FOXA2 protein degradation in vitro. Taken together, all the studies demonstrate that FOXA2 mediated by TRIM36 promotes CRC progression by inhibiting the Nrf2/GPX4 ferroptosis signaling pathway, thus providing a new therapeutic target for CRC treatment.

## Graphical abstract

FOXA2 deletion contributed to tumor suppression and chemoresistance in CRC by inducing ferroptosis via the Nrf2/GPX4 signaling suppression. TRIM36 is identified as a suppressor of FOXA2 by inducing its K48‐linked polyubiquitination, which may have a therapeutic benefit in CRC patients with FOXA2 overexpression.

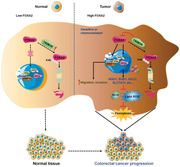

## 1 Introduction

Colorectal cancer (CRC), a common gastrointestinal malignancy, is the third most diagnosed tumor worldwide.^[^ ^1^ ^]^ Presently, marked progress has been achieved for CRC treatments such as surgery, radiotherapy, and chemotherapy, but the 5‐year overall survival rates for patients with CRC are still unsatisfactory and less than 60%, ^[^ ^2^ ^]^ which is associated with its postoperative recurrence, chemoresistance, and metastasis.^[^ ^3^ ^]^ Herein, finding effective biomarkers and/or targets is imperative to explore the mechanisms underlying CRC progression for developing more efficient therapeutic strategies.

Ferroptosis is a recently identified new form of regulated cell death that is characterized by iron accumulation, lipid peroxidation, and reduction of antioxidant GSH levels, and is different with apoptosis, autophagy, and necroptosis.^[^ ^4^ ^]^ Growing studies demonstrate that ferroptosis can be triggered through the suppression of cystine/glutamate transporter (SLC7A11/xCT) activation and GPX4.^[^ ^5^ ^]^ Interestingly, the GPX4 levels in tumor specimens of patients with advanced CRC are markedly higher than the ones in para‐carcinoma tissues.^[^ ^6^ ^]^ Growing evidence has also revealed that ferroptosis induction through promoting intracellular Fe^2+^ levels and ROS production, depressing GPX4, and decreasing GSH contents in CRC cells significantly contributes to the suppression of CRC in the clinic.^[^ ^7^, ^8^ ^]^ In contrast, restraining ferroptosis gives rise to tumor progression and drug resistance in CRC treatment.^[^ ^9^ ^]^ Furthermore, OXA, as a first‑line chemotherapy for CRC, was recently shown to induce ferroptosis and oxidative stress in CRC cells by suppressing Nrf2 signaling pathway.^[^ ^10^ ^]^ Other chemotherapies such as propofol, Honokiol and cisplatin are also reported to induce ferroptotic cell death in vitro and in vivo, which are alternative strategies for CRC treatment.^[^ ^11^, ^12^, ^13^ ^]^ On the basis of these studies, driving ferroptosis in CRC cells may provide effective treatment strategies for CRC‐targeted therapy.

The forkhead box A (FOXA) gene family of transcription factors is composed of three members, including FOXA1, FOXA2, and FOXA3, which are encoded by individual genes in animal cells.^[^ ^14^ ^]^ The FOXA gene family plays an essential regulatory role in embryonic development, immune system, the cell cycle, cell longevity, and energy metabolism homeostasis.^[^ ^15^, ^16^ ^]^ FOXA2 (also known as hepatocyte nuclear factor 3 beta HNF3β), a member of the FOXA transcription factor family, has 3 exons and 2 introns and a conserved DNA binding domain. FOXA2 can bind to the promoter and enhancer regions of target genes, thereby mediating their transcription.^[^ ^17^ ^]^ For instance, FOXA2 has been identified to bind to the promoter region of CRE‐binding protein (CREB) and hepatocyte nuclear factor 6 (HNF6) and then activate their transcription.^[^ ^18^ ^]^ FOXA2 is widely expressed in many types of tissues and organs, such as the pancreas, liver, lung, heart, and other tissues, and participates in cell growth, differentiation, death, metabolism, and inflammatory response.^[^ ^19^, ^20^, ^21^ ^]^ However, increasing studies have observed the functional relevance of FOXA2 in tumor growth. Abnormal expression of FOXA2 is associated with the processes of proliferation, epithelial mesenchymal transition (EMT), and metastasis of numerous types of tumors, such as hepatocellular carcinoma (HCC), prostate cancer, gastric cancer, and breast cancer.^[^ ^19^, ^22^, ^23^, ^24^ ^]^ For example, FOXA2 could maintain the stemness of ovarian cancer cells, and its reduction decreased the formation of tumor spheres.^[^ ^24^ ^]^ Additionally, FOXA2 could be induced by the active Wnt/β‐catenin signaling pathway, which was related to the invasive phenotype of primary prostate cancer.^[^ ^25^ ^]^ Recently, emerging evidence demonstrated that FOXA2 elevation was associated with colon cancer progression through regulating cell proliferation, EMT process, and apoptosis.^[^ ^26^ ^]^ However, the function and molecular mechanisms of FOXA2 are not fully studied in CRC, such as ferroptosis and chemoresistance. Considering the potential of FOXA2 during the development of CRC, FOXA2 may be a very valuable hallmark and drug target for CRC.

In the present study, we further explored the role of FOXA2 in drug‐sensitive and ‐resistant CRC cell lines and in the constructed xenograft mouse model, AOM/DSS‐induced CAC tumorigenesis using the intestinal‐specific FOXA2‐knockout mice, and the Apc^Min/+^ mouse model with CRC to uncover the involved molecular mechanisms. Our findings not only revealed a novel link between FOXA2 and ferroptosis but also disclosed a novel mechanism of OXA resistance, suggesting that FOXA2 is a predictive factor with therapeutic potential for CRC treatment.

## 2 Results

### 2.1 FOXA2 Expression is Up‐Regulated in CRC Patients

**Figure 1:**
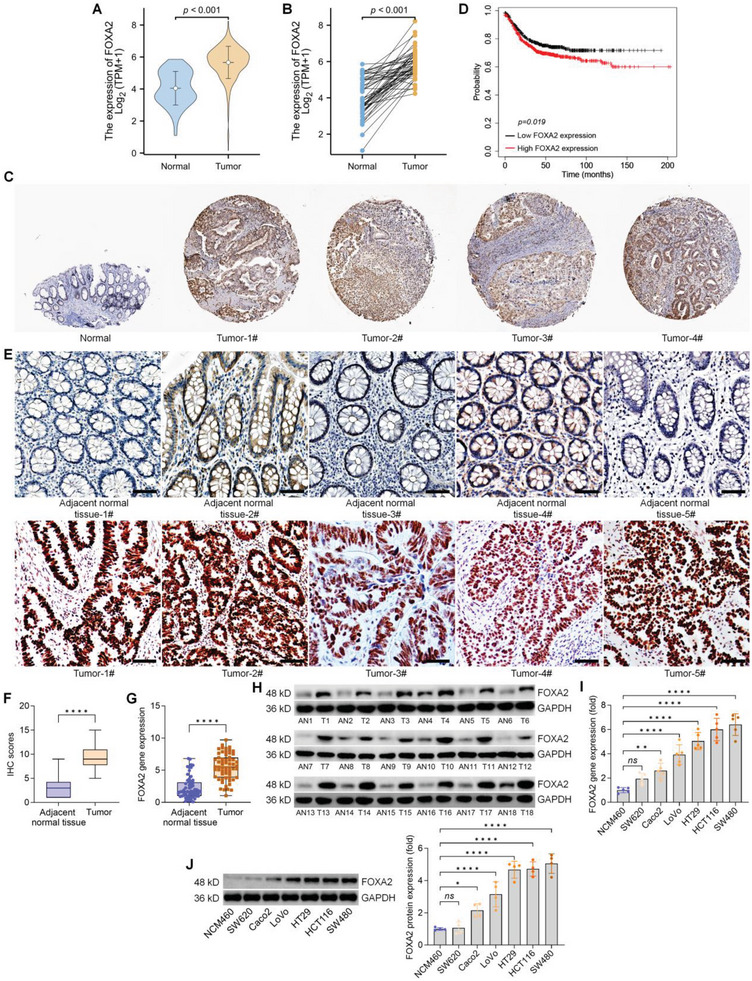
FOXA2 expression is up‐regulated in CRC patients. A,B) FOXA2 expression profile in CRC tissues from TCGA cohort. C) Representative IHC images for FOXA2 in CRC tissues and normal tissues (https://www.proteinatlas.org/). D) Kaplan‐Meier analysis for relapse‐free‐survival (RFS) of CRC patients with high (*n* = 709) or low (*n* = 633) FOXA2 expression based on the median expression of FOXA2 from the Kaplan‐Meier Plotter (https://kmplot.com/analysis/index.php?p = service). E) Images of IHC staining for FOXA2 in CRC tissues and the paired adjacent normal tissues from our cohort. Scale bar = 120 µm. F) IHC scores of FOXA2 expression levels in paired normal and CRC samples were quantified. G) RT‐qPCR analysis for FOXA2 gene expression in human CRC tissues and the matched adjacent normal tissues (ANT) from our cohort (*n* = 60). H) FOXA2 protein expression in eighteen paired CRC tissues was examined using western blot. I) RT‐qPCR (*n* = 5) and J) western blot (*n* = 4) assays for FOXA2 gene and protein expression levels in six CRC cell lines and non‐tumor cell line NCM460. Data are marked as the means ± SD. ^*^ *p* < 0.05, ^**^ *p* < 0.01, ^****^ *p* < 0.0001.

By analyzing the TCGA database, Metascape analysis showed that six transcription factors (ELF3, FOXA2, HNF1A, SP1, JUN, and HNF4A) were associated with the regulation of the functions of DEGs during CRC progression (Figure S1A, Supporting Information). Among these factors, the potential of FOXA2 on CRC was almost uninvestigated. The results of PPI network analysis by the STRING database demonstrated that FOXA2 might be associated with cell proliferation‐ and EMT‐related signals such as TP53, Fibronectin (FN1), and SMADs (Figure S1B, Supporting Information). To further assess the functions of FOXA2 in CRC, we first investigated FOXA2 expression changes in CRC samples using the TCGA database. We found that FOXA2 expression was significantly increased in CRC tissues compared with the normal samples (**Figure** **1**A,B). According to the Human Protein Atlas online database, 1 of 12 patients exerted medium expression of FOXA2, 3 among 12 patients displayed low expression, and 8 of 12 patients showed no significant expression in patients with CRC (Figure 1C). Survival information from the online KM‐Plotter database for relapse‐free‐survival (RFS) showed that CRC patients with high FOXA2 levels experienced markedly worse clinical outcomes (Figure 1D), while no significant difference was detected in the changes of overall survival rates among CRC patients based on TCGA database (Figure S1C and Table S3, Supporting Information). Our hospital cohort confirmed that FOXA2 expression was strongly up‐regulated in tumor tissues from CRC patients compared with the paired adjacent normal samples by IHC, RT‐qPCR, and western blot analysis (Figure 1E‐H). Furthermore, CRC cell lines, particularly HCT‐116 and SW480, exerted markedly increased expression of FOXA2 at mRNA and protein levels in comparison to the nontumor cell line NCM460 (Figure 1I,J). These results initially illustrated that FOXA2 up‐regulation was involved in CRC growth.

### 2.2 FOXA2 Enhances the Proliferation, Migration, and Invasion of CRC Cells in Vitro or in Vivo

**Figure 2:**
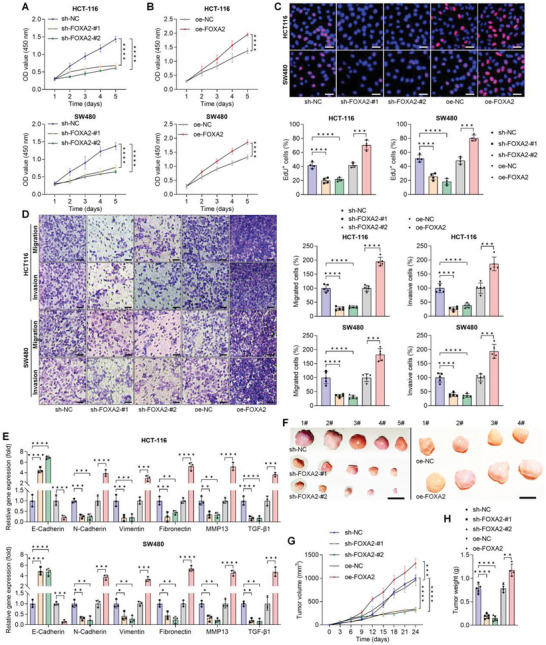
FOXA2 enhances the proliferation, migration and invasion of CRC cells in vitro or in vivo. CCK8 analysis for cell proliferation of HCT‐116 and SW480 cells with FOXA2 A) knockdown or B) over‐expression (*n* = 4). C) EdU staining of HCT‐116 and SW480 cells transfected with sh‐FOXA2 or oe‐FOXA2 as shown (*n* = 4). Scale bar = 50 µm. D) Transwell analysis for CRC cell migration and invasion after FOXA2 knockdown or over‐expression (*n* = 5). Scale bar = 50 µm. E) RT‐qPCR analysis for EMT markers in HCT‐116 and SW480 cells after FOXA2 knockdown or over‐expression (*n* = 3). F) Tumor samples from the indicated groups of mice expressing the control vector, sh‐FOXA2 or oe‐FOXA2 plasmids (*n* = 5 or 4 in each). G) Tumor formation volume and H) tumor weights were measured. Data are marked as the means ± SD. ^*^ *p* < 0.05, ^**^ *p* < 0.01, ^***^ *p* < 0.001, ^****^ *p* < 0.0001.

Due to the potential role of FOXA2 in CRC progression, its expression was then deleted and promoted by transfecting with the constructed sh‐FOXA2 and FOXA2 over‐expression plasmids, respectively. RT‐qPCR and western blot assays confirmed the substantial transfection efficacy in HCT‐116 and SW480 cells (Figure S1D‐G, Supporting Information). Results by CCK‐8 and EdU staining showed that FOXA2 knockdown significantly reduced the proliferation of CRC cell lines, while its overexpression markedly promoted the proliferative capacity of CRC cells (**Figure** **2**A‐C). Transwell assay in Figure 2D indicated that transcription of sh‐FOXA2 strongly impaired the migration and invasion properties of HCT‐116 and SW480 cells, but oe‐FOXA2 plasmid significantly strengthened the number of migrated and invaded CRC cells. Accordingly, the expression levels of EMT hallmarks, including E‐cadherin, N‐cadherin, Vimentin, Fibronectin, MMP13, and TGF‐β1 were examined by RT‐qPCR. As shown in Figure 2E, increased E‐Cadherin and decreased N‐cadherin, Vimentin, Fibronectin, MMP13, and TGF‐β1 were detected in CRC cells with FOXA2 deletion compared with the sh‐NC group; however, opposite expression trends were observed in HCT‐116 and SW480 cells over‐expressing FOXA2. Thereafter, xenograft animal models with CRC were then established by injection of HCT‐116 cells with stable FOXA2 knockdown or over‐expression. As expected, sh‐FOXA2 effectively reduced the tumor size and growth rates compared with the sh‐NC group, but promoting FOXA2 expression significantly facilitated the tumor growth in mice (Figure 2F,G), accompanied with much higher tumor weights (Figure 2H). Western blot results showed evidently reduced FOXA2 expression in tumor tissues from sh‐FOXA2 mice. Additionally, stronger FOXA2 was detected in tumor samples collected from oe‐FOXA2 mice than that of the oe‐NC group (Figure S2A, Supporting Information), which was confirmed by IHC staining (Figure S2B,C, Supporting Information). Furthermore, KI‐67, a marker of tumor cell proliferation, and Fibronectin were also found to be highly down‐regulated by FOXA2 ablation, while being promoted in tumor tissues with FOXA2 over‐expression. These findings suggested that FOXA2 could enhance CRC cell proliferation, migration, and invasion in vitro and in vivo.

### 2.3 Positive Correlation Between FOXA2 and Nrf2/GPX4 Signaling in CRC Cell Lines

**Figure 3:**
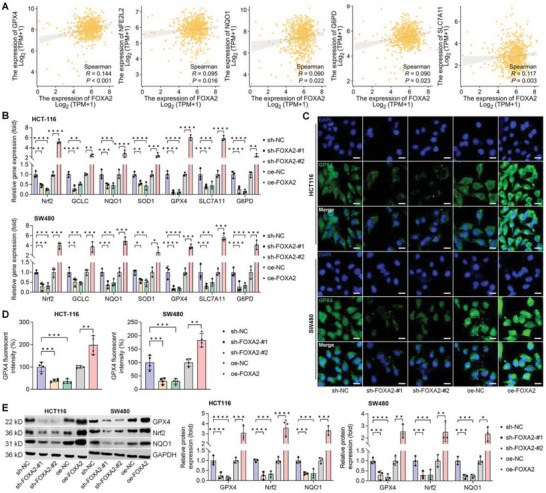
Positive correlation between FOXA2 and Nrf2/GPX4 signaling in CRC cell lines. A) Positive correlation between FOXA2 expression and GPX4, NFE2L2 (Nrf2), NQO1, G6PD, and SLC7A11 in CRC patients from TCGA database. B) RT‐qPCR analysis for genes including Nrf2, GCLC, NQO1, SOD1, GPX4, SLC7A11 and G6PD in HCT‐116 and SW480 cells with FOXA2 knockdown or over‐expression (*n* = 3). C,D) IF staining for GPX4 expression in CRC cell lines transfected with sh‐FOXA2 or oe‐FOXA2 (*n* = 4). Scale bar = 20 µm. E) Western blot analysis for GPX4, Nrf2, and NQO1 protein expression levels in FOXA2‐diminished or ‐over‐expressed HCT‐116 and SW480 cells (*n* = 3). Data are marked as the means ± SD. ^*^ *p* < 0.05, ^**^ *p* < 0.01, ^***^ *p* < 0.001, ^****^ *p* < 0.0001.

RNA‐Seq analysis was then conducted to examine the gene expression in sh‐NC and sh‐FOXA2 HCT‐116 cells to explore the underlying mechanisms revealing the oncogenic potential of FOXA2 in CRC cells. The volcano plot showed the DEGs regulated by FOXA2 knockdown. From the volcano plot result, significantly down‐regulated genes associated with EMT, including MMP13, FN1, and CDH2 (N‐Cadherin), were observed, accompanied by increased CDH1 (also known as E‐Cadherin). Notably, oxidative stress‐ and ferroptosis‐related DEGs such as NFE2L2 (also known as Nrf2), NQO1, SOD1, GCLM, GPX4, SLC7A11, ACACA and ACSL4 were detected in HCT‐116 cells after FOXA2 knockdown (Figure S3A, Supporting Information). The GO results and KEGG pathway analysis revealed the top biological and pathological pathways most influenced by FOXA2 deletion, including responses to oxidative stress and ferroptosis (Figure S3B,C, Supporting Information). In addition, GSEA analysis confirmed that the DEGs were enriched in the Nrf2 pathway (Figure S3D, Supporting Information). Transcription factor‐target analysis indicated that many overlapped DEGs were mediated by factors in the TRRUST database using Metascape, including NFE2L2 (Figure S3E, Supporting Information). Subsequently, we extracted PPI and MCODE components to investigate the correlations between FOXA2 and CRC in detail. The list of identified genes was shown in Figure S3F,G, Supporting Information, which included three models enriched in cellular responses to stress‐, NFE2L2 (Nrf2) survival signaling‐ and oxidative stress response‐associated signaling pathways. KEGG analysis identified a ferroptosis signaling pathway within many of these pathways mediated by FOXA2 knockdown, as displayed in Figure S3H, Supporting Information. Subsequently, the correlations between the FOXA2 and ferroptosis‐related markers were explored in CRC patients by the TCGA database. As shown in **Figure** **3**A, significant positive correlations between FOXA2 gene expression and GPX4, NFE2L2 (Nrf2), NQO1, G6PD, and SLC7A11 were detected by Spearman analysis, revealing the potentially negative effects of FOXA2 on ferroptotic cell death in CRC. To confirm our hypothesis, these genes were examined using HCT‐116 and SW480 cells with FOXA2 knockdown or overexpression. As expected, RT‐qPCR results indicated that FOXA2 deletion significantly reduced the mRNA expression levels of Nrf2, GCLC, NQO1, SOD1, GPX4, SLC7A11, and G6PD in CRC cell lines, while its over‐expression led to the opposite result of these genes (Figure 3B). The decreased expression of GPX4, Nrf2, and NQO1 induced by sh‐FOXA2 was validated by IF staining and western blot assays in CRC cells, but oe‐FOXA2 resulted in significant increases of GPX4, Nrf2 and NQO1 (Figure 3C‐E). IHC staining confirmed the promotive effects of FOXA2 on GPX4 and Nrf2, as indicated by the obviously enhanced positive expression of GPX4 and Nrf2 in tumor samples collected from oe‐FOXA2 mice (Figure S4A,B, Supporting Information). Together, these findings demonstrated that FOXA2 could mediate ferroptotic cell death, which was involved in CRC progression.

### 2.4 FOXA2 Suppression Induces Ferroptosis in CRC Cell Lines

**Figure 4:**
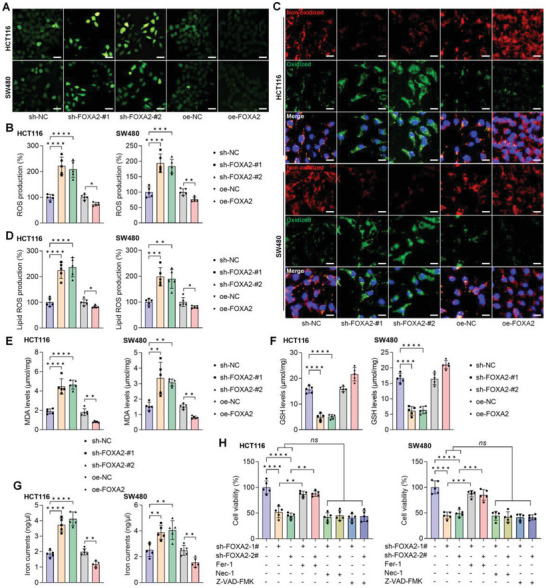
FOXA2 suppression induces ferroptosis in CRC cell lines. A,B) ROS production was measured by DCF‐DA staining in CRC cells with FOXA2 knockdown or over‐expression. Scale bar = 50 µm. C,D) Lipid ROS generation in HCT‐116 and SW480 cells was measured by C11‐BODIPY581/591 staining post‐sh‐FOXA2 or oe‐FOXA2 plasmid transfection. Scale bar = 20 µm. E) MDA levels, (F) GSH contents and G) iron currents in the show groups of CRC cells were examined. H) HCT‐116 and SW480 cells with FOXA2 knockdown were treated with ferroptosis inhibitor (Fer‐1, 1 µM), necroptosis inhibitor (Nec‐1, 10 µM), or apoptosis inhibitor (Z‐VAD‐FMK, 10 µM) for another 24 h. Then, all cells were collected for cell viability examination using CCK‐8 analysis. Data are marked as the means ± SD (*n* = 5 in each). ^**^ *p* < 0.01, ^***^ *p* < 0.001, ^****^ *p* < 0.0001; *ns*, no significant difference.

Given that ferroptosis is triggered by the impairment of cellular redox balance and GSH has a key to eliminate lipid ROS accumulation.^[^ ^4^, ^5^ ^]^ Subsequently, we examined whether sh‐FOXA2 and oe‐FOXA2 was associated with ROS production and GSH synthesis. First, DCF‐DA staining showed that FOXA2 ablation strongly induced ROS generation in CRC cells, while lower ROS was detected in cells over‐expressing FOXA2 than in those of the oe‐NC group (**Figure** **4**A,B). Consistently, significant lipid ROS accumulation was identified in HCT‐116 and SW480 cells with FOXA knockdown compared with the sh‐NC group; however, CRC cells over‐expressing FOXA2 exerted markedly reduced lipid ROS by C11‐BODIPY581/591 staining (Figure 4C,D). Lipid oxidation was also evaluated by assessing cellular MDA levels. Similarly, FOXA2 deletion strongly increased MDA levels in HCT‐116 and SW480 cells, but lower MDA contents were detected in CRC cells transfected with the oe‐FOXA2 plasmid (Figure 4E). On the contrary, GSH levels were evidently down‐regulated after transfection with sh‐FOXA2 in CRC cells, whereas over‐expressing FOXA2 led to a significant increase in GSH (Figure 4F). To verify these observations, iron contents were examined, which is another primary cause of ferroptosis.^[^ ^4^, ^5^, ^6^ ^]^ As shown in Figure 4G, markedly enhanced iron currents were detected in HCT‐116 and SW480 cells with FOXA2 knockdown; however, FOXA2 overexpression reduced their levels. Finally, to examine whether other types of cell death were involved in FOXA2 knockdown‐inhibited CRC cell proliferation, ferroptosis, necroptosis, and apoptosis inhibitors, including Fer‐1, Nec‐1, and Z‐VAD‐FMK, respectively, were subjected to HCT‐116 and SW480 cells with or without sh‐FOXA2 transfection. CCK‐8 results showed that sh‐FOXA2‐reduced cell viability was significantly rescued upon Fer‐1 addition, while Nec‐1 and Z‐VAD‐FMK treatments had no influence on the changes in cell viability interfered by sh‐FOXA2 (Figure 4H), indicating that FOXA2 deletion‐restrained CRC proliferation might be largely through the process of ferroptosis induction.

### 2.5 FOXA2 Suppression Sensitizes CRC Cells to OXA Treatment by Facilitating Ferroptosis

**Figure 5:**
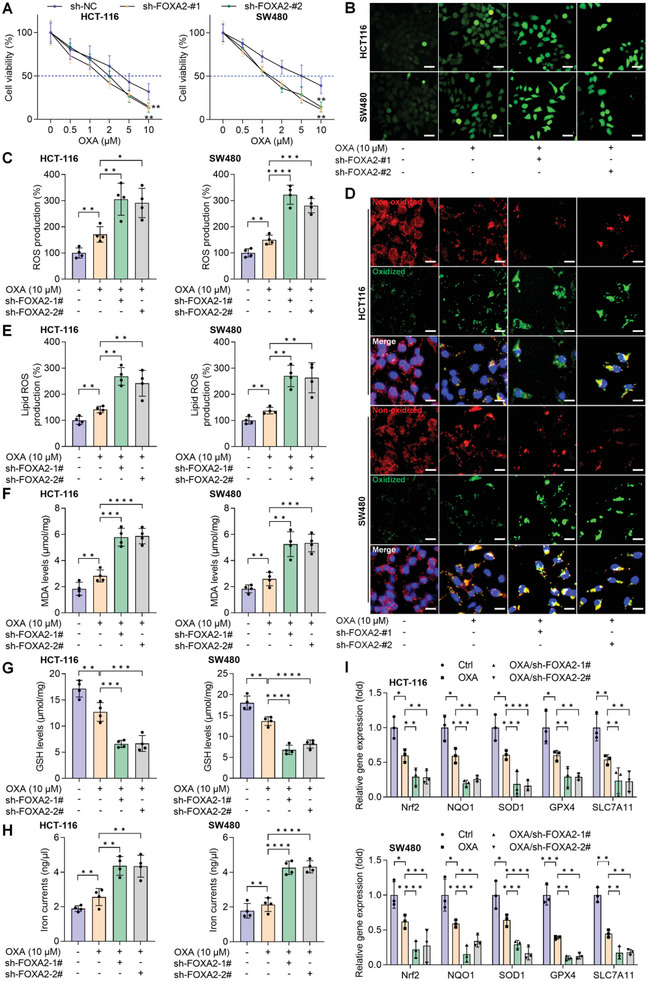
FOXA2 suppression sensitizes CRC cells to OXA treatment by facilitating ferroptosis. HCT‐116 and SW480 cells were transfected with sh‐FOXA2, followed by OXA treatments for another 24 h. Then, all cells were harvested for subsequent assays. A) CCK‐8 analysis for cell viability evaluation (*n* = 4). B,C) DCF‐DA staining was used to examine ROS production (*n* = 4). Scale bar = 50 µm. D,E) C11‐BODIPY581/591 staining was conducted for the measurements of lipid ROS production (*n* = 4). Scale bar = 20 µm. F) MDA contents, G) GSH levels, and H) iron currents were assessed (*n* = 4). I) Ferroptosis hallmarks including Nrf2, NQO1, SOD1, GPX4 and SLC7A11 were calculated by RT‐qPCR (*n* = 3). Data are marked as the means ± SD. ^*^ *p* < 0.05, ^**^ *p* < 0.01, ^***^ *p* < 0.001, ^****^ *p* < 0.0001.

To explore whether FOXA2 had a role in ferroptosis to mediate chemosensitivity and resistance in CRC, OXA, recommended as a first‐line therapeutic drug for clinical metastatic CRC, was subjected to HCT‐116 and SW480 cells with or without FOXA2 absence. CCK‐8 analysis demonstrated that OXA‐reduced cell viability of CRC cells was further down‐regulated by sh‐FOXA2 (**Figure** **5**A). Notably, cellular ROS production, lipid ROS accumulation, and MDA levels were up‐regulated by OXA exposure in CRC cells, and these processes were markedly facilitated upon FOXA2 knockdown (Figure 5B‐F). Furthermore, sh‐FOXA2 dramatically strengthened the function of OXA to reduce GSH contents in HCT‐116 and SW480 cells (Figure 5G), accompanied by elevated iron levels (Figure 5H). In line with these observations, the mRNA expression levels of Nrf2, NQO1, SOD1, GPX4, and SLC7A11 retarded by OXA were further decreased by sh‐FOXA2 (Figure 5I). In vivo animal studies showed similar results that stable FOXA2 knockdown significantly facilitated the anti‐cancer role of OXA, as evidenced by the smaller tumor size, growth rates, and tumor weights (Figure S5A‐C, Supporting Information). IHC staining validated the much lower expression of KI‐67, Fibronectin, GPX4, and Nrf2 in tumor samples from the OXA/sh‐FOXA2 group of mice, which was comparable with the OXA and sh‐FOXA2 single treatment groups (Figure S5D, Supporting Information). These above findings elucidated that FOXA2 suppression might sensitize CRC cells to OXA treatment by promoting ferroptotic cell death.

### 2.6 FOXA2 knockdown Induces Ferroptosis in Chemoresistant CRC Cell Lines

**Figure 6:**
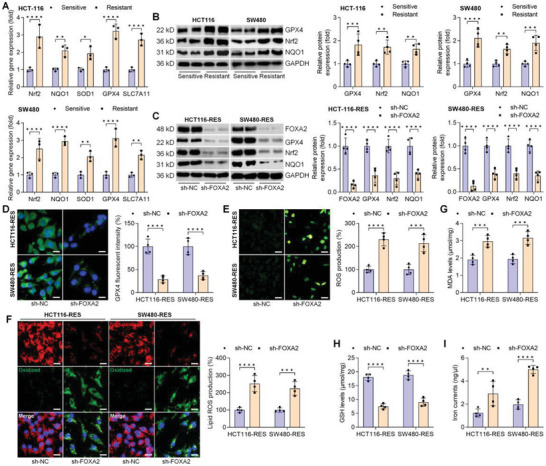
FOXA2 knockdown induces ferroptosis in chemoresistant CRC cell lines. A) RT‐qPCR analysis for Nrf2, NQO1, SOD1, GPX4 and SLC7A11 gene expression levels in drug‐sensitive or ‐resistant HCT‐116 and SW480 cells (*n* = 3). B) Western blot assay for GPX4, Nrf2, and NQO1 protein expression levels in HCT‐116 and SW480 cells with or without chemoresistance (*n* = 4). C) Western blot assay for FOXA2, GPX4, Nrf2, and NQO1 protein expression levels in chemoresistant HCT‐116 and SW480 cells with or without FOXA2 knockdown (*n* = 4). (D) GPX4 expression by IF staining in drug‐resistant CRC cells after transfection with sh‐FOXA2 (*n* = 4). Scale bar = 20 µm. E) ROS and (F) lipid ROS production by DCF‐DA (Scale bar = 50 µm) and C11‐BODIPY581/591 (Scale bar = 20 µm) staining, respectively, in chemoresistant HCT‐116 and SW480 cells with FOXA2 knockdown (*n* = 4). G) MDA levels, H) GSH contents, and I) iron currents in drug‐resistant HCT‐116 and SW480 cells after FOXA2 knockdown (*n* = 4). Data are marked as the means ± SD. ^*^ *p* < 0.05, ^**^ *p* < 0.01, ^***^ *p* < 0.001, ^****^ *p* < 0.0001.

In the region, CRC cell lines with chemoresistance, including HCT‐116‐RES and SW480‐RES, were constructed by long‐term OXA exposure to deeply explore the effects of FOXA2 on drug resistance‐associated CRC in vitro. As shown in Figure S6A,B, Supporting Information, we unexpectedly found a stronger FOXA2 expression in OXA‐resistant CRC cell lines than that of the cells with chemosensitivity. Similarly, higher gene and/or expression levels of Nrf2, NQO1, SOD1, GPX4, and SLC7A11 were observed in HCT‐116 and SW480 cells with OXA resistance, which were comparable with the sensitive groups (**Figure** **6**A,B). Thereafter, FOXA2 expression was inhibited in chemoresistant CRC cells to further explore its influence on CRC progression with drug resistance in vitro. Western blot analysis showed that FOXA2, GPX4, Nrf2, and NQO1 expression levels were obviously diminished in HCT‐116‐RES and SW480‐RES cells by transfecting with sh‐FOXA2 (Figure 6C). IF staining confirmed GPX4 down‐regulation by sh‐FOXA2 in OXA‐resistant CRC cell lines, as indicated by the weaker fluorescence (Figure 6D). Additionally, HCT‐116‐RES and SW480‐RES cells exerted stronger cellular ROS production and lipid ROS accumulation after sh‐FOXA2 transfection when compared with the sh‐NC group (Figure 6E,F). As expected, FOXA2 deletion significantly increased MDA levels and iron currents in OXA‐resistant CRC cells, along with evidently decreased GSH contents (Figure 6G‐I). Furthermore, we found that FOXA2 knockdown could markedly improve the sensitivity of chemoresistant CRC cells to OXA treatments, as indicated by the decreased cell viability and the number of EdU‐positive cells (Figure S6C,D, Supporting Information). Results from transwell assay supported the function of sh‐FOXA2 to repress the migration and invasion of drug‐resistant CRC cell lines after OXA exposure (Figure S6E, Supporting Information). Together, the data above revealed that FOXA2 suppression could improve the sensitivity of chemoresistant CRC cells to OXA treatments for CRC suppression by reducing the cell proliferative, migratory, and invasive capacities and inducing ferroptosis.

### 2.7 FOXA2 Knockdown CRC Cells are Sensitive to OXA‐Induced Ferroptosis Via the Nrf2 Activation

**Figure 7:**
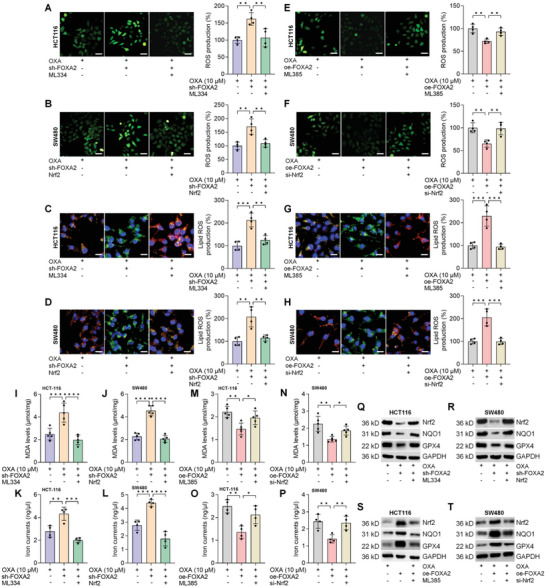
FOXA2 knockdown CRC cells are sensitive to OXA‐induced ferroptosis via the Nrf2 activation. HCT‐116 cells with FOXA2 knockdown or over‐expression were incubated with OXA (10 µM) alone or combination with Nrf2 activator (ML334, 20 µM) or inhibitor (ML385, 5 µM) for an additional 24 h. SW480 cells co‐transfected with sh‐FOXA2 and Nrf2 plasmids, or oe‐FOXA2 and si‐Nrf2 were exposed to OXA (10 µM) treatment for another 24 h. Then, all HCT‐116 and SW480 cells were harvested for studies as follows. A‐H) DCF‐DA (Scale bar = 50 µm) and C11‐BODIPY581/591 (Scale bar = 20 µm) staining were performed to examine ROS and lipid ROS production in CRC cells treated as shown (*n* = 4). I,J) MDA levels in HCT‐116 and SW480 cells were examined. K,L) Examination of iron currents in CRC cells. (M,N) Calculation for cellular MDA levels. O,P) Iron currents in CRC cells were assessed (*n* = 5). Q‐T) Western blot analysis for Nrf2, NQO1, and GPX4 protein expression levels in CRC cells were conducted (*n* = 3). Data are marked as the means ± SD. ^*^ *p* < 0.05, ^**^ *p* < 0.01, ^***^ *p* < 0.001, ^****^ *p* < 0.0001.

Given the potential role of Nrf2 in FOXA2‐mediated CRC growth and ferroptosis, its expression was then promoted or depressed in chemo‐sensitive HCT‐116 or SW480 cells with or without FOXA2 expression in the presence of OXA. To facilitate Nrf2 expression, its activator (ML334) or over‐expression plasmid was subjected to CRC cells. Western blot analysis confirmed the up‐regulation of Nrf2 and its down‐streaming signal NQO1 induced by ML334 in HCT‐116 cells (Figure S6A, Supporting Information). Transfection with the Nrf2 plasmid also led to a significant increase in Nrf2 gene and protein expression levels in SW480 cells (Figure S6B, Supporting Information). After Nrf2 inhibitor ML385 exposure, markedly weakened Nrf2 and NQO1 expression was detected in HCT‐116 cells (Figure S6C, Supporting Information). Similarly, Nrf2 silence was efficiently triggered by si‐Nrf2 transfection compared with the si‐NC group in SW480 cells (Figure S6D, Supporting Information). As shown in **Figure** **7**A–D, sh‐FOXA2‐enhanced cellular and lipid ROS production was strongly diminished upon Nrf2 expression promotion induced by ML334 or the Nrf2 plasmid in OXA‐treated CRC cells. However, FOXA2‐eliminated ROS levels and lipid ROS accumulation provoked by OXA were regained upon Nrf2 suppression by ML385 or si‐Nrf2 (Figure 7E–H). What's more, ML334 and Nrf2 almost abrogated FOXA2 knockdown‐increased MDA and iron contents in OXA‐incubated CRC cells (Figure 7I‐L). In contrast, oe‐FOXA2‐caused reduction of MDA and iron levels in OXA‐treated CRC cells were markedly rescored upon Nrf2 signal suppression (Figure 7M‐P). Consistently, Nrf2, NQO1, and GPX4 protein expression levels inhibited by sh‐FOXA2 were obviously abolished by ML334 addition or Nrf2 plasmid transfection (Figure 7Q,R). However, Nrf2 signaling blockage by ML385 or si‐Nrf2 clearly reduced FOXA2‐caused up‐regulation of NQO1 and GPX4 in CRC cells after OXA exposure (Figure 7S,T). In line with the results above, ML334 or Nrf2 plasmid transfection markedly eliminated the function of sh‐FOXA2 to restrain CRC cell proliferation (Figure S8A–D, Supporting Information). Furthermore, in OXA‐treated HCT‐116 and SW480 cells, FOXA2 overexpression‐caused higher proliferative capacity was markedly ameliorated upon ML385 and si‐Nrf2 treatment (Figure S8E–H, Supporting Information). Collectively, these data elucidated that FOXA2 depressed OXA‐triggered ferroptosis through the activation of the Nrf2 pathway.

### 2.8 RSL3 Promotes the Sensitivity of FOXA2‐Overexpressed and Chemoresistant CRC Cells to OXA Treatment

**Figure 8:**
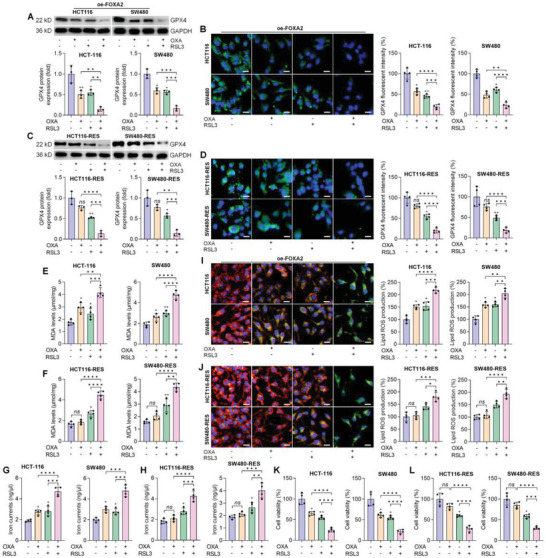
RSL3 promotes the sensitivity of FOXA2‐overexpressed and chemoresistant CRC cells to OXA treatment. Drug‐sensitive CRC cells with FOXA2 over‐expression or chemoresistant CRC cells were subjected to OXA (10 µM), GPX4 inhibitor (RSL3, 100 nM) single or double incubation for another 24 h. Next, all cells were harvested for studies as following. A‐D) Western blot and IF staining analysis for GPX4 protein expression levels in cells treated as shown (*n* = 3 or 4 in each). E,F) MDA levels and G,H) iron contents were examined (*n* = 4). I,J) Lipid ROS production was measured using C11‐BODIPY581/591 staining (*n* = 4). K,L) Cell viability of the shown CRC cells with or without chemoresistance was examined using CCK‐8 analysis (*n* = 4). Data are marked as the means ± SD. Scale bar = 20 µm. ^+^ *p* < 0.05, ^++^ *p* < 0.01 versus the Ctrl group; ^*^ *p* < 0.05, ^**^ *p* < 0.01, ^***^ *p* < 0.001, ^****^ *p* < 0.0001; *ns*, no significant difference.

Because GPX4 is particularly involved in ferroptotic cell death and is a key transcriptional target gene of Nrf2.^[^ ^4^, ^5^, ^36^ ^]^ Then, the GPX4 inhibitor RSL3 was subjected to HCT‐116 and SW480 cells to further explore if ferroptosis participated in the drug sensitivity of CRC cells to OXA. Western blot and IF staining results showed that simultaneous exposure of OXA and RSL3 markedly reduced the GPX4 protein expression levels in HCT‐116 and SW480 cells transfected with oe‐FOXA2 plasmids (**Figure** **8**A,B). Intriguingly, RSL3 co‐addition improved the sensitivity of HCT‐116‐RES and SW480‐RES cells to OXA incubation, as proved by the markedly decreased expression of GPX4 (Figure 8C,D). MDA levels and iron contents triggered by OXA were strongly enhanced by RSL3 co‐incubation in FOXA2‐overexpressing CRC cells. Similarly, GPX4 suppression by RSL3 also promoted the sensitivity of chemoresistant CRC cells to OXA incubation for ferroptosis induction, as indicated by the up‐regulated MDA and iron levels (Figure 8E‐H). Consistent with the observation above, lipid ROS production irritated by OXA was further enhanced by RSL3 in drug‐sensitive HCT‐116 and SW480 cells with FOXA2 over‐expression (Figure 8I). Of note, GPX4 repression by RSL3 remarkably induced lipid ROS accumulation in OXA‐treated CRC cells with chemoresistance (Figure 8J). CCK‐8 analysis finally confirmed that oe‐FOXA2 CRC cells with co‐treatments of OXA and RSL3 exerted significantly decreased cell viability compared with the single group (Figure 8K). Furthermore, RSL3 addition markedly improved the chemoresistant HCT‐116 and SW480 cells to OXA treatment for cell proliferation suppression (Figure 8L). Taken together, GPX4 inhibition by RSL3 promoted the sensitivity of oe‐FOXA2 and drug‐resistant CRC cells to OXA.

### 2.9 Identifying a Potent Suppressor of FOXA2

**Figure 9:**
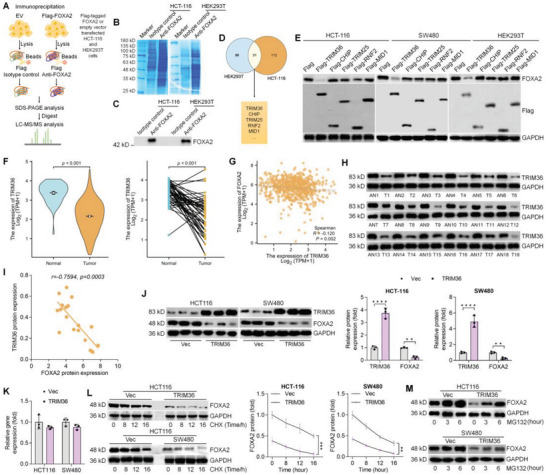
Identifying a potent suppressor of FOXA2. A) Working model of the protocols used to identify potent binding proteins and suppressors of FOXA2. B) SDS‐PAGE band showing the immunoprecipitated proteins in HCT‐116 and HEK293T cells with anti‐FOXA2 antibody *vs* the isotype‐controlled IgG. C) Immunoblotting assay to validate the presence of FOXA2 in the immunoprecipitated samples. D) There were a total of 31 overlapping FOXA2 binding proteins identified both in HCT‐116 and HEK293T cells, including the listed E3 ligases TRIM36, CHIP, TRIM25, RNF2, and MID1. E) Western blot indicating the FOXA2 expression in HCT‐116 and HEK293T cells transfected with the shown plasmids. F) TRIM36 expression profile in CRC tissues from TCGA cohort. G) Correlation between FOXA2 and TRIM36 from TCGA database. H) TRIM36 protein expression in eighteen paired CRC tissues was examined using western blot. I) Spearman's correlation between TRIM36 and FOXA2 protein expression in the eighteen CRC patients. J) Western blot analysis for TRIM36 and FOXA2 in CRC cells transfected with TRIM36 plasmids. (K) RT‐qPCR analysis for FOXA2 in CRC cells with or without TRIM36 over‐expression. L) FOXA2 protein expression levels in empty vector and TRIM36‐overexpressed HCT‐116 or SW480 cells were examined at the shown time after CHX (20 µg ml^−1^) incubation. M) Western blot indicating the influence of MG132 (10 µM) on FOXA2 expression levels in CRC cells with or without TRIM36 over‐expression. Data are marked as the means ± SD. ^**^ *p* < 0.01, ^****^ *p* < 0.0001.

The HEK293T cell line has been widely used as the model cell in studies of plasmid transfection and exploration of interactions between proteins.^[^ ^37^, ^38^ ^]^ To further explore how FOXA2 was involved in CRC, IP and mass spectrometry (MS) analysis were conducted to investigate FOXA2 binding proteins using HCT‐116 and HEK293T cells stably transfected with FOXA2 (**Figure** **9**A,B). Western blotting assay validated the presence of FOXA2 in the anti‐FOXA2‐IP sample, revealing the successful FOXA2 pull‐down by IP (Figure 9C). There were 31 overlapping proteins associated with FOXA2, including 5 E3 ligases (TRIM36, CHIP, TRIM25, RNF2, and MID1) between the 2 cell lines (Figure 9D). FOXA2 has been reported to be ubiquitinated for its protein degradation.^[^ ^39^ ^]^ E3 ligases can regulate the polyubiquitination of substrates in cells and are involved in cellular signaling, proliferation, cell death, protein stability control, EMT, and carcinogenesis.^[^ ^40^ ^]^ Therefore, we attempted to explore which one of these identified E3 ligases might be particularly important in the modulation of FOXA2 stability and expression during CRC progression. Western blot results in Figure 9E showed that only TRIM36 resulted in FOXA2 degradation in HCT‐116, SW480, and HEK293T cells. The results using the TCGA database further indicated that the mRNA expression of TRIM36 was markedly lower in tumor tissues than that in normal tissues (Figure 9F). Spearman's correlation results indicated that the expression of TRIM36 was negatively correlated with the expression of FOXA2 on the basis of the TCGA dataset (Figure 9G). In line with the TCGA assay results, CRC tumor tissues from our cohort exerted lower TRIM36 protein expression than the paired adjacent normal tissues (Figure 9H). Consistently, a significantly negative correlation between TRIM36 and FOXA2 protein expression levels was observed among CRC patients (Figure 9I). We further found that TRIM36 over‐expression markedly decreased the protein level of FOXA2 in CRC cell lines (Figure 9J), but had no significant influence on the gene expression levels of FOXA2 (Figure 9K), revealing that TRIM36 might affect FOXA2 stability. To verify the assumption, the protein synthesis inhibitor CHX and the proteasome inhibitor MG132 were subjected to TRIM36‐overexpressing HCT‐116 and SW480 cells. The western blot results showed that promoting TRIM36 evidently shortened the half‐life of endogenous FOXA2 protein in HCT‐116 and SW480 cells following CHX exposure (Figure 9L). Additionally, FOXA2 expression was modestly up‐regulated in TRIM36‐overexpressing CRC cells with MG132 incubation (Figure 9M). Taken together, these above findings demonstrated that TRIM36 might have a negative correlation with FOXA2 via a ubiquitin‐proteasome pathway to regulate the stability of FOXA2.

### 2.10 TRIM36 Interacts with FOXA2 and Induces its K48‐Linked Polyubiquitination

**Figure 10:**
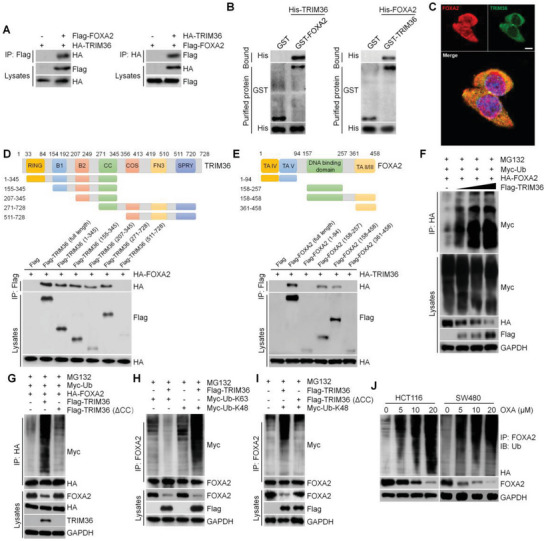
TRIM36 interacts with FOXA2 and induces its K48‐linked polyubiquitination. A) Co‐IP analysis of HEK293T cells transfected with Flag‐tagged FOXA2 and HA‐tagged TRIM36. Anti‐Flag and anti‐HA antibodies were used for western blot assay. B) GST precipitation indicating the direct interaction of TRIM36 with FOXA2 using purified GST‐FOXA2 and His‐tagged TRIM36 (left) or purified GST‐TRIM36 and His‐FOXA2 (right) by western blot analysis. GST was defined as a control. C) IF images of HEK293T cells co‐transfected with 24 h of Flag‐tagged FOXA2 (red) and HA‐tagged TRIM36 (green). Scale bar = 15 µm. D,E) Schematic indicating full‐length and truncated TRIM36 (top) and FOXA2 (top) with representative Co‐IP assays (bottom) for the mapping analysis of the domains responsible for the TRIM36/FOXA2 interaction in HEK293T cells. F) Lysates of HEK293T cells transfected with plasmids expressing HA‐FOXA2, Myc‐Ub and increasing amounts of Flag‐TIRM36 were immunoprecipitated with anti‐HA beads and immunoblotted using an anti‐Myc antibody. G) Western blots showing FOXA2 ubiquitination in HEK293T cells transfected with the indicated plasmids in different combinations. ∆CC, deletion of the CC domain. H) Western blot analysis showing K48 ubiquitination of FOXA2 in HEK293T cells co‐transfected with Flag‐TRIM36 and Myc‐K48‐Ub or Myc‐K63‐Ub. I) Western blot analysis showing K48 ubiquitination of FOXA2 in HEK293T cells co‐transfected with Flag‐TRIM36, Flag‐TRIM36 (ΔCC), and Myc‐K48‐Ub. (J) FOXA2 protein expression levels and its ubiquitination levels in 24 h of OXA‐treated HCT‐116 and SW480 cells.

In this section, we further substantiated the potentially specific interaction between TRIM36 and FOXA2 using HEK293T, HCT‐116, and SW480 cells. Co‐IP and GST analysis in **Figure** **10**A,B showed that TRIM36 directly interacted with FOXA2 in HEK293T cells, which were identified in colon cancer cells (Figure S9A,B). Additionally, double IF staining showed an obvious colocalization for TRIM36 and FOXA2 in HEK293T, HCT‐116 and SW480 cells (Figure 10C and Figure S9C, Supporting Information). Molecular mapping results indicated that the CC domain (residues 271–345) was the main fragment of TRIM36 responsible for binding to FOXA2 at the DNA binding domain (residues 157–257) (Figure 10D,E). Similar findings were observed in HCT‐116 and SW480 cells (Figure S9D,E, Supporting Information). Given that TRIM36 is an E3 ligase and FOXA2 can be ubiquitinated,^[^ ^39^, ^41^ ^]^ we subsequently investigated if TRIM36 influenced FOXA2 stability through ubiquitination. As shown in Figure 10F and Figure S9F, Supporting Information, we observed that TRIM36 enhanced the polyubiquitination of FOXA2 via a dose‐dependent manner in colon cancer cells. Of note, the CC domain mutation (ΔCC) abolished the function of TRIM36 to induce FOXA2 ubiquitination in HEK293T, HCT‐116, and SW480 cells (Figure 10G and Figure S9G, Supporting Information). We further found that TRIM36 decreased the K63‐linked ubiquitination but increased the K48‐linked ubiquitination of FOXA2 in cells (Figure 10H and Figure S9H, Supporting Information), while being arrogated upon CC domain mutation (Figure 10I and Figure S9I, Supporting Information). Finally, in HCT‐116 and SW480 cells, higher FOXA2 ubiquitination was induced by OXA treatments in a dose‐dependent manner, accompanied by lower FOXA2 stability (Figure 10J). Taken together, all these data suggested that TRIM36 could directly interact with FOXA2, and induce its K48‐linked polyubiquitination for its protein degradation in colon cancer cells.

### 2.11 Conditional Knockout of FOXA2 in IECs Ameliorates Colitis‐Associated Tumorigenesis in Vivo

**Figure 11:**
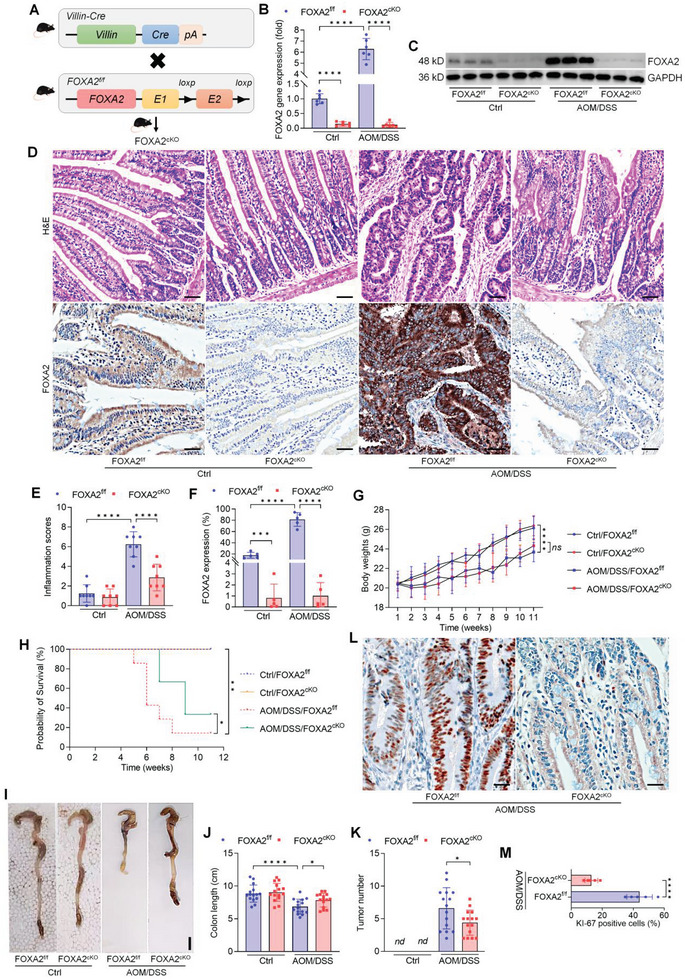
Conditional knockout of FOXA2 in IECs ameliorates colitis‐associated tumorigenesis in vivo. A) Scheme protocols of recombination in *Villin‐Cre*; FOXA2*^f/f^* mice. B,C) RT‐qPCR and western blot assays for FOXA2 gene and protein expression levels in colon crypts from the mice treated with or without AOM/DSS (*n* = 6). D) H&E and IHC staining of FOXA2 in colon from FOXA2*^f/f^* and FOXA2^cKO^ mice (n = 8). Scale bar = 120 µm. E) Inflammation scores were quantified. (F) FOXA2 expression by IHC staining was calculated. G) Body weights of mice were recorded (*n* = 10–15 in each group). H) Overall survival rates for each group of mice (*n* = 10–15 in each group). I) Images for colons from all groups of mice. J) Colon length was measured (*n* = 15). (K) Tumor number on colon was examined (*n* = 15). L) IHC staining for KI‐67 in colon tissues was performed (*n* = 5). Scale bar = 50 µm. Data are marked as the means ± SD. ^*^ *p* < 0.05, ^**^ *p* < 0.01, ^***^ *p* < 0.001, ^****^ *p* < 0.0001; *ns*, no significant difference.

To confirm the role of FOXA2 in CRC, we established a colitis‐associated CRC mouse model using intestinal epithelial cell‐specific FOXA2 knockout (FOXA2^cKO^) mice (**Figure** **11**A). AOM/DSS was used to generate colitis‐associated cancer.^[^ ^30^ ^]^ Mice were identified to harbor the epithelial FOXA2 deletion by RT‐qPCR and western blot assays (Figure 11B,C). Furthermore, significantly elevated FOXA2 expression levels were detected in colon tissues of AOM/DSS‐treated mice compared with the Ctrl group. Histological analysis by H&E staining of the colon tissues from AOM/DSS‐treated mice showed severer inflammation and tumorigenesis, but the mice with FOXA2^cKO^ displayed markedly ameliorated histological colonic damage (Figure 11D‐F). Body weight loss was detected in mice receiving AOM/DSS treatment compared with the Ctrl group, while conditional knockout of FOXA2 had no significant influence on the body weight changes among all groups of animals (Figure 11G). Importantly, FOXA2^cKO^ mice were more susceptible to AOM/DSS‐triggered colitis‐associated colon tumorigenesis, as confirmed by the improvement in survival ratio, increase in colon length, and reduction in tumor numbers (Figure 11H‐K). Consistently, KI‐67 staining by IHC revealed that tumor cell proliferation was significantly ameliorated in FOXA2^cKO^ mouse colons (Figure 11L). Double IF staining finally suggested that mice with FOXA2 absence exerted lower GPX4 and Nrf2 positive expression in tumor areas of AOM/DSS‐treated colons (Figure S10, Supporting Information). Together, all the above data confirmed that FOXA2 deletion in IECs could attenuate colitis‐associated tumorigenesis in vivo.

### 2.12 FOXA2 Ablation in ApcMin/+ Ameliorates Spontaneous Intestinal Tumor Burden

Finally, another CRC mouse model was established by using Apc^Min/+^ mice to further investigate the effects of FOXA2 on CRC progression. As shown in Figure S11A‐C, Supporting Information, higher FOXA2 mRNA and protein expression levels were observed in the colon tissues of Apc^Min/+^ mice than those of WT mice. We then examined the biological function of FOXA2 and its contribution during CRC tumorigenesis by knocking down FOXA2 using intravenous injection of AAV (Figure S11D,E, Supporting Information). Significantly decreased expression of FOXA2 was identified in the colon tissues of AAV‐shFOXA2/Apc^Min/+^ mice compared with the AAV‐shCtrl group (Figure S11F,G, Supporting Information). IHC and H&E staining showed that FOXA2 knockdown evidently ameliorated tumorigenesis in the colon of Apc^Min/+^ mice (Figure S11H, Supporting Information). Additionally, Apc^Min/+^ mice with FOXA2 deficiency showed markedly improved overall survival rates (Figure S11I, Supporting Information). Importantly, obviously reduced tumor number was observed in AAV‐shFOXA2/Apc^Min/+^ mice compared with the shCtrl group (Figure S11J,K, Supporting Information), accompanied by clearly decreased expression of KI‐67 positive cells (Figure S11L, Supporting Information). Western blot results in Figure S11M, Supporting Information, further confirmed evidently mitigated protein expression levels of GPX4, Nrf2, and HO‐1 in colon tissues of AAV‐shFOXA2/Apc^Min/+^ mice (Figure S11M, Supporting Information). Collectively, these data identified the suppressive effects of shFOXA2 on CRC tumorigenesis through restraining Nrf2/GPX4 signaling.

## 3 Discussion

CRC is a common malignant tumor of the digestive system, but its therapeutic outcome is still unsatisfactory across the world due to a poor understanding of the mechanisms for its tumorigenesis and/or associated chemoresistance.^[^ ^1^, ^2^, ^3^ ^]^ Thus, searching for a novel prognostic marker and efficient target site is still a hot spot for improving CRC treatment. Accumulating studies have identified that transcription factors exert crucial roles in the regulation of gene expression, and are considered as potential drug targets for numerous diseases.^[^ ^42^ ^]^ FOXA2 is a member of the FOXA family of the Forkhead box transcription factors and has been shown to be an essential marker involved in the development of various types of tumors.^[^ ^19^, ^22^, ^23^, ^24^, ^25^, ^26^ ^]^ In the present study, we found significantly elevated FOXA2 expression levels in tumor samples of CRC patients compared to those of the normal group, which was in line with previous observations,^[^ ^26^ ^]^ and its high expression predicted poor RFS in patients with CRC by KM analysis. We similarly found that FOXA2 knockdown markedly reduced the proliferative, migratory, and invasive properties of CRC cells in vitro and efficiently restrained tumor growth in vivo by using various CRC mouse models. Surprisingly, we identified a positive correlation between FOXA2 and Nrf2/GPX4 signaling in patients with CRC. We newly found higher expression of FOXA2, Nrf2, and GPX4 in OXA‐resistant CRC cells than those in the OXA‐sensitive group. Correspondingly, cellular ROS generation, lipid peroxidation, accumulation of MDA contents and iron levels, and GSH reduction were strongly induced by sh‐FOXA2 both in drug‐sensitive and ‐resistant CRC cells, eventually contributing to ferroptosis through retarding the expression of Nrf2/GPX4 signaling, which was accompanied by decreased cell proliferation. FOXA2 has been recognized to be ubiquitinated to control its protein expression and degradation.^[^ ^39^ ^]^ We finally found that the down‐regulation of E3 ligase TRIM36 during CRC progression might largely contribute to the K48‐linked polyubiquitination of FOXA2, thereby resulting in FOXA2 degradation and the subsequent effects on CRC growth. A negative correlation between TRIM36 and FOXA2 expression was identified in CRC patients. Multiple analysis results confirmed that TRIM36 could directly interact with FOXA2. Taken together, all our findings elucidated that FOXA2 suppression by TRIM36 represents a potential therapeutic strategy for CRC treatment by inducing ferroptosis via withholding Nrf2/GPX4 signaling pathway.

FOXA2, as a transcription factor, belongs to the forkhead/winged‐helix family and has been suggested to suppress tumor growth in cervical cancer and ameliorate EMT in HCC to control metastasis.^[^ ^22^ ^]^ However, FOXA2 also functions as an oncogene to facilitate tumorigenesis. For instance, FOXA2 is a driving factor in promoting neuroendocrine prostate cancer.^[^ ^43^ ^]^ Additionally, FOXA2 suppression is involved in afatinib‐inhibited stemness and tumor spheres of pancreatic cancer stem cells.^[^ ^44^ ^]^ Thus, the effects of FOXA2 on tumorigenesis are complicated without a thorough understanding, which may be associated with the different types of cancer cells. EMT is a program of epithelial cells to obtain a mesenchymal‐like phenotype, which can limit total surgical resection and result in therapeutic resistance, ultimately causing tumor recurrence.^[^ ^45^ ^]^ Methods to restrain the EMT process are effective for the suppression of invasion and metastasis of numerous types of tumor cells, including CRC.^[^ ^46^ ^]^ Recently, FOXA2 was initially identified to play an oncogene role in CRC by facilitating EMT and metastasis.^[^ ^26^ ^]^ Consistently, we provided further support that FOXA2 up‐regulation was indeed involved in CRC progression. Briefly, knockdown of FOXA2 expression overtly inhibited proliferation and EdU‐positive CRC cells. However, FOXA2 overexpression markedly facilitated the proliferative capacity of CRC cells. Additionally, injection of stable FOXA2 knockdown or over‐expression CRC cells into BALB/c nude mice further supported the effects of FOXA2 on tumor growth in vivo. Similarly, AOM/DSS‐treated mice harboring the epithelial FOXA2 knockout (FOXA2^cKO^) showed significantly mitigated CAC tumorigenesis with higher overall survival rates. In line with these data, Apc^Min/+^ mice with FOXA2 knockdown by tail vein injection of AAV‐shFOXA2 strongly reduced spontaneous intestinal tumor burden. These data elucidated that FOXA2 has a tumor‐promoting role in the progression of CRC.

The EMT process is marked by numerous proteins such as E‐Cadherin, N‐Cadherin, Vimentin, Fibronectin, MMPs, and TGF‐β, etc. Among these molecules, E‐Cadherin is a pivotal epithelial marker regulating the inhibitory effects on tumor metastasis. However, proteins including N‐Cadherin, Vimentin, Fibronectin, and TGF‐β are essential promoters involved in EMT formation and metastasis.^[^ ^47^ ^]^ Recently, FOXA2 expression was reported to be positively correlated with epithelial marker genes, such as E‐cadherin and Zinc finger E‐box Binding homeobox 2 (ZEB2), thereby inhibiting EMT in breast cancer cells.^[^ ^48^ ^]^ FOXA2 was sufficient for sustaining epithelial characteristics, and withstood TGF‐β‐caused cell migration and transcriptomic alterations in lung cancer cells, and acted as an inhibitor of tumor metastasis via suppressing EMT.^[^ ^49^ ^]^ On the contrary, our study detected a different observation that EMT process was greatly restrained in CRC cells with FOXA2 knockdown, as indicated by the markedly decreased migration and invasion. But overexpressing FOXA2 obviously promoted the migratory and invasive capacities of CRC cells, accompanied by the decreased E‐cadherin, and increased N‐cadherin, Vimentin, Fibronectin, MMP13, and TGF‐β. These in vitro results were in line with a recent study concluding that EMT of colon cancer can be suppressed by down‐regulating FOXA2 with a decrease in E‐cadherin and Vimentin.^[^ ^26^ ^]^ Therefore, we assumed that the regulatory effects of FOXA2 on EMT and metastasis events are largely dependent on the different types of cancer cells, thus requiring more studies for specific tumor types.

FOXA2 has been recognized as an anti‐apoptosis factor in numerous tumor cells, including HCC and CRC cells.^[^ ^22^, ^26^ ^]^ But in gastric cancer, FOXA2 could induce apoptotic cell death to perform its anti‐tumor functions.^[^ ^50^ ^]^ Different from apoptosis, ferroptosis is a recently identified form of regulated cell death that is accompanied by the accumulation of lipid ROS derived from iron metabolism.^[^ ^4^, ^5^, ^6^ ^]^ Growing studies recognize the potential role of ferroptosis in tumor translational medicine, including overcoming drug resistance and progression prevention.^[^ ^51^ ^]^ Homeostatic malfunction of ferroptosis is considered to be a key reason for chemoresistance, and thus it is all‐important to explore how to improve the sensitivity of tumor cells to chemotherapies by inducing ferroptosis in the clinic.^[^ ^52^ ^]^ Ferroptosis has been implicated in the occurrence, progression, and treatment of CRC. For example, cetuximab is a monoclonal antibody that targets the epidermal growth factor receptor (EGFR), and can induce ferroptotic cell death by GPX4 inhibitor RSL3 through depressing Nrf2/HO‐1 pathway in CRC cells with KRAS mutation.^[^ ^53^ ^]^ OXA, a first‐line chemotherapy drug approved for CRC or advanced CRC worldwide, can provoke ferroptosis in CRC by suppressing the Nrf2 signaling pathway.^[^ ^10^, ^54^ ^]^ But OXA resistance emergence is also a challenging factor responsible for the failure of CRC treatment.^[^ ^27^ ^]^ In our study, we unexpectedly observed that FOXA2 expression was positively correlated with GPX4, Nrf2, NQO1, and SLC7A11 in CRC patients from the TCGA database. Furthermore, FOXA2 knockdown markedly reduced the expression of these molecules in HCT‐116 and SW480 cells, along with increased cellular ROS production, lipid ROS accumulation, MDA levels, and iron currents, but decreased GSH contents. However, opposite results of these phenomena were observed in CRC cells with FOXA2 over‐expression. Bioinformatic analysis supported these findings that ferroptosis signaling pathway was among the top by KEGG in the CRC cells with sh‐FOXA2 treatment. These findings demonstrated that FOXA2 suppression‐restrained CRC progression was partially attributed to ferroptosis induction. Importantly, higher FOXA2 expression was detected in OXA‐resistant CRC cells than in chemo‐sensitive ones, accompanied by up‐regulated Nrf2 and GPX4 expression levels. We found that FOXA2 overexpression accelerated the resistance to OXA‐triggered ferroptosis through weakening ROS generation, MDA levels, iron and lipid peroxidation accumulation. Conversely, FOXA2 deletion remarkably improved the sensitivity of CRC cells to OXA treatment via promoting ferroptosis. Together, our results confirmed that ferroptosis had a key role in the anti‐cancer efficiency of OXA. Furthermore, we recently discovered that FOXA2 negatively regulated ferroptosis during OXA treatment.

Nrf2 is a major modulator of the antioxidant response, which contributes to its anti‐ferroptotic activity by regulating its target genes, such as NQO1, GCLC, and SLC7A11.^[^ ^55^ ^]^ Increasing studies have noticed that Nrf2 signaling suppression could enhance the sensitivity of tumor cells to ferroptosis, while its activation contributed to ferroptosis resistance.^[^ ^56^, ^57^ ^]^ In addition, GPX4, the only reported enzyme and a downstream target gene of Nrf2, can be capable of directly lessening complex phospholipid hydroperoxides. Herein, targeting GPX4 may be an efficient approach to induce ferroptosis.^[^ ^36^, ^54^, ^58^ ^]^ More recently, FOXA2 was reported to mediate Nrf2 target genes in fumarate hydratase‐deficient cells, and its knockdown decreased the expression of Nrf2 target genes, thus altering anti‐oxidant associated metabolites.^[^ ^59^ ^]^ Similar influence of sh‐FOXA2 on Nrf2 signaling was observed in our current study. Targeting Nrf2 for its suppression by si‐Nrf2 or its inhibitor ML385 in FOXA2‐overexpressed CRC cells restored ROS levels, MDA contents, iron accumulation, and lipid peroxidation production. In contrast, promoting Nrf2 markedly abolished the capacity of sh‐FOXA2 to induce ferroptosis in OXA‐treated CRC cells. Meanwhile, targeting GPX4 with its suppressor RSL3 strongly triggered ferroptosis in CRC cells with FOXA2 overexpression or chemoresistance, contributing to cell proliferation reduction. These observations illustrated that FOXA2‐mediated ferroptosis was closely associated with the activation of Nrf2/GPX4 axis. However, there was a still limitation in how FOXA2 exerts its regulatory role in Nrf2, whether in a direct or indirect manner. As for this, further research is still needed in the near future.

Ubiquitination is a reversible posttranslational modification process that can target proteins for degradation and mediate the function of proteins.^[^ ^60^ ^]^ Ubiquitin itself has seven lysines (K), and every lysine is able to be further conjugated to another ubiquitin molecule at its carboxyl terminus, forming diverse types of polyubiquitin chains. The K48‐ and K63‐linked polyubiquitin chains are recognized as the dominating types of ubiquitin linkage. Polyubiquitination mediated by K48‐linked targets proteins for proteasomal degradation, while K63‐linked polyubiquitination has an important role in regulating cell signaling.^[^ ^61^ ^]^ FOXA2 has been reported to be modified by ubiquitination. Metabolite pyocyanin results in the posttranslational modifications and degradation of FOXA2 by inducing its polyubiquitination.^[^ ^62^ ^]^ Neural precursor cells expressed developmentally downregulated 4‐like (NEDD4L), an E3 ubiquitin protein ligase, can restrain FOXA2 expression also by the ubiquitination modification manner, which is involved in osteogenic differentiation of bone marrow‐derived mesenchymal stem cells (BMSCs).^[^ ^63^ ^]^ Smad ubiquitylation regulatory factor‐1 (Smurf1) is also an E3 ubiquitin ligase, and has been shown to regulate the Lys48‐linked poly‐ubiquitination of FOXA2, thereby resulting in FOXA2 degradation in the proteasome, which is closely associated with the invasion and migration of cervical cancer cells.^[^ ^64^ ^]^ We then sought to identify endogenous regulators of FOXA2 expression in cells during CRC progression by using IP and mass spectrometry analysis, and in doing so, the E3 ligase TRIM36 was identified as an essential inhibitor of FOXA2. TRIM36 plays a key role in mediating the stability and function of p53 protein, herein influencing the therapeutic efficacy of gastric cancer.^[^ ^65^ ^]^ TRIM36 was also reported to be markedly decreased in some lung cancer cell lines, breast cancer, and esophageal cancer.^[^ ^41^, ^66^ ^]^ An in vitro study suggested that TRIM36 could induce cyclin E ubiquitination, which was involved in the suppression of HCC.^[^ ^67^ ^]^ In our present study, we further found that TRIM36 expression was significantly down‐regulated in tumor tissues from patients with CRC compared with the normal samples and had a negative correlation with FOXA2. Moreover, promoting TRIM36 expression obviously reduced the protein expression of FOXA2. Cell studies further showed that TRIM36 could bind and directly interact with FOXA2 and induce the K48‐linked polyubiquitination of FOXA2, thereby promoting its protein degradation. What's more, mutation of the CC domain obviously diminished the function of TRIM36 to induce K48‐linked FOXA2 polyubiquitination. Finally, we found that OXA dose‐dependently reduced FOXA2 protein expression levels in CRC cell lines, accompanied by higher FOXA2 ubiquitination, which may subsequently let CRC cells undergo ferroptosis. Our present work highlighted a previously undescribed molecular mechanism for the regulation of FOXA2 stability.

In conclusion, our study confirmed that FOXA2 up‐regulation was involved in CRC progression. FOXA2 deletion contributed to tumor suppression and chemoresistance in CRC by inducing ferroptosis via the Nrf2/GPX4 signaling suppression. Our data provided a new insight into the molecular mechanism of the effect of FOXA2 on ferroptosis and chemotherapy for CRC. Furthermore, TRIM36 was identified as a suppressor of FOXA2 by inducing its K48‐linked polyubiquitination, which may have a therapeutic benefit in CRC patients with FOXA2 overexpression. Collectively, our study contributed to a better understanding of CRC tumorigenesis and revealed that FOXA2 restrained by TRIM36 might be a novel therapeutic target for CRC treatment through inducing ferroptosis.

## 4 Experimental Section

### Reagent and antibodies

The apoptosis inhibitor Z‐VAD‐FMK (#HY‐16658B), necroptosis inhibitor necrostatin‐1 (Nec‐1, #HY‐15760), oxaliplatin (OXA, #HY‐17371), cycloheximide (CHX, #HY‐12320) and proteasome inhibitor MG132 (#HY‐13259) were obtained from MedChemExpress (MCE, Shanghai, China). Azoxymethane (AOM, #A5486), dextran sulfate sodium (DSS, #265152), Nrf2 inhibitor (#SML1833), and Nrf2 activator ML334 (#5059870001) were obtained from Sigma Aldrich (Louis, MO, USA). Ferroptosis inducer RSL3 (#S8155) and inhibitor Fer‐1 (#S7243) were purchased from Selleck Chemical (Shanghai, China). The following primary antibodies were used: anti‐FOXA2 antibody (#NBP2‐02088, 1:1000), anti‐NQO1 antibody (#NB200‐209, 1:1000), and anti‐GPX4 antibody (#NBP2‐75511, 1:1000) were obtained from Novus Biologicals (Littleton, CO, USA); anti‐Nrf2 antibody (#12721, 1:1000), anti‐KI‐67 antibody (#34330, 1:1000), anti‐Flag antibody (#14793), anti‐HA antibody (#5017), anti‐Myc antibody (#2276) and anti‐GAPDH (#5174) were obtained from Cell Signaling Technology (Beverly, MA, USA); anti‐Fibronectin antibody (#MA5‐11981, 1:1000), and anti‐TRIM36 antibody (#PA5‐28401, 1:1000) were purchased from Thermo Fisher Scientific (Waltham, MA, USA). Anti‐GFP (#ab290), and secondary antibodies including Rabbit Anti‐Mouse IgG H&L (HRP) (#ab6728), Goat Anti‐Rabbit IgG H&L (HRP) (#ab6721), Goat Anti‐Rabbit IgG H&L (Alexa Fluor 488) (#ab150077) and Goat Anti‐Mouse IgG H&L (Alexa Fluor® 647) (#ab150115) were obtained from Abcam (Cambridge, UK).

### Clinical Specimens

All human studies were approved by the ethics committee of the Shandong Cancer Hospital and Institute, Shandong First Medical University, and Shandong Academy of Medical Sciences (Jinan, Shandong PR, China), and the study was conducted following the principles of the Declaration of Helsinki. Informed consent was obtained from all patients prior to the study. A total of 60 primary CRC tissues and the matched adjacent normal colon epithelium tissues were collected from the surgical samples clinically and histologically diagnosed with CRC (from 2013 to 2018) by the Shandong Cancer Hospital and Institute, Shandong First Medical University and Shandong Academy of Medical Sciences, and these patients received no chemotherapy or radiotherapy before surgery. The information on patients’ clinicopathological conditions is provided in Table S1, Supporting Information.

### Cells and Treatments

Human CRC cell lines including HCT‐116, LoVo, HT‐29, CaCo2, SW480, and SW620 were purchased from American Type Culture Collection (ATCC, USA). A normal human colon mucosal epithelial cell line NCM460 was obtained from the Type Culture Collection of the Chinese Academy of Sciences (Shanghai, China). Human HEK293T cell line was purchased from Cell Bank, Type Culture Collection, Chinese Academy of Sciences (CBTCCCAS, Shanghai, China). OXA‐resistant HCT‐116 (HCT‐116‐RES) and SW480 (SW480‐RES) cells were induced from HCT‐116 and SW480 cells, respectively, as previously described.^[^ ^27^ ^]^ Briefly, HCT‐116 and SW480 cells were exposed to the medium containing increasing OXA concentrations, and finally, HCT‐116‐RES and SW480‐RES cells resistant to 10 µM of OXA were screened. All cells were maintained in DMEM (Gibco, Carlsbad, CA, USA) supplemented with 10% fetal bovine serum (FBS, Gibco), 100 U mL^−1^ penicillin, and 100 µg mL^−1^ streptomycin (Gibco) at 37 °C in an incubator containing 5% CO_2_. There was no mycoplasma contamination detected.

### In Vitro Transfection

To establish the stable FOXA2‐knockdown, FOXA2‐overexpression, Nrf2‐overexpression, and TRIM36‐overexpression CRC cell lines, the human lentivirus‐sh‐FOXA2, FOXA2 overexpression (oe‐FOXA2) plasmids, Nrf2 and TRIM36 plasmids were purchased from GeneChem (Shanghai, China). Nrf2 specific small interference RNAs (siNrf2) were synthesized by RiboBio (Guangzhou, China). Transfection was strictly conducted using Lipofectamine3000 (#L3000015; Thermo Fisher Scientific) in accordance with the manufacturer's protocol. In brief, when cells were grown to ≈60%–80% confluence, 1 µg construct and 1 µl Lipofectamine 3000 were added to the culture media. Cells were collected for further analysis 48 h after transfection. Stably transfected CRC cell lines were selected with 2 µg ml^−1^ puromycin (#HY‐B1743A; MCE) and obtained after continuous screening for one week post‐virus infection or construct transfection.

### Plasmids Construction

The full‐length and truncated sequences for human cDNA of FOXA2, TRIM36, CHIP, TRIM25, RNF2, and MID1 were amplified and sub‐cloned into the p3xFlag‐Myc‐CMV24 (#E6151; SEEBIO BIOTECH Co., Ltd., Shanghai, China) and/or pcDNA‐HA vectors (#128034; Addgene, Ke Lei Biological Technology Co., Ltd, Shanghai, China). Plasmids encoding full‐length GST‐FOXA2 or GST‐TRIM36 were obtained by cloning the cDNA of FOXA2 or TRIM36 into the pGEX‐4T‐1‐GST vectors (#129567, Addgene). Ubiquitin and its derivatives were cloned into the Myc‐tagged pcDNA5 vectors (Invitrogen, Thermo Fisher Scientific).

### RNA Isolation and RT‐qPCR

Total RNA from tissues or cells was extracted using the TRIzol reagent (#9108; TaKaRa, Osaka, Japan). RT‐qPCR was conducted with a PrimeScript RT Reagent Kit (#RR036A, TaKaRa) and a SYBR Premix Ex Taq (#RR620A, TaKaRa) following the manufacturer's protocols. Glyceraldehyde‐3‐phosphate dehydrogenase (GAPDH) was used as the loading control. Gene expression data were analyzed using the 2^−∆∆^ *^Ct^* method. The sequences for specific primers were listed in Table S2, Supporting Information.

### Intracellular ROS Detection

ROS production in cells was measured using 2’,7’‐dichlorofluorescein diacetate (DCFH‐DA) (#S0033M; Beyotime Biotechnology) following the manufacturer's instructions. Briefly, cells after treatments were added with the DCFH‐DA (10 µmol l^−1^) reagent and incubated at 37 °C for 20 min. After washing with a serum‐free medium, ROS production was analyzed using a fluorescent microscope (ZEISS Axio Observer A1, Germany).

### Lipid ROS Assay

CRC cells were seeded on 12‐well plates (20 000 cells per well). After treatments, the cells were stained with 10 µM of C11‐BODIPY 581/591 (#GC40165; Glpbio, Shanghai, China) and incubated at 37 °C for 30 min following the manufacturer's protocols. After washing, images were captured using a fluorescence microscope (ZEISS Axio Observer A1, Germany). The alteration of lipid peroxidation was standardized and exhibited by the ratio of green fluorescence to red fluorescence.

### Biochemical Parameters Examination

MDA, a biomarker of lipid peroxidation, in cells was measured using an MDA assay kit (#A003‐4‐1; Nanjing Jincheng Bioengineering Institute, Nanjing, China) according to the manufacturer's protocols. The GSH concentration in cell lysates was examined using an assay kit (#A006‐2) also from Nanjing Jiancheng Bioengineering Institute following the manufacturer's instructions. The luminescence of the yellow product of the reaction was read at 405 nm and normalized by protein concentration detected with a BCA Kit (#P0012; Beyotime Biotechnology). The iron contents were measured using an Iron Assay Kit (#MAK025; Sigma) following the manufacturer's instructions. In brief, CRC cells (2 × 10^6^) were homogenized in iron assay buffer and then centrifuged at 15,000 × g for 10 min at 4 °C. The insoluble material was then discarded. For total iron, 50 µl cellular samples were added to 96‐well plate, and the volume was increased to 100 µl each well using the iron assay buffer. Next, 5 µl of iron reducer was added to each well to reduce Fe^3+^ to Fe^2+^. Samples were subsequently mixed with a horizontal shaker and incubated for 30 min at 25 °C in dark. Next, 100 µl of Iron Probe was subjected to samples and incubated at 25 °C for another 1 h in the dark. Finally, the absorbance at 593 nm was read on a microplate reader (Molecular Devices).

### Immunohistochemistry (IHC)

IHC analysis was carried out as previously suggested with a slight modification.^[^ ^28^ ^]^ In brief, tissues were fixed in 4% paraformaldehyde and cut into 5 um thick slices, followed by embedding in paraffin, dewaxing using xylene, and rehydration through graded concentrations of ethanol. Then, a citrate antigen repair solution (Sangon Biotech, Shanghai, China) was used and antigen retrieval was performed by a microwave oven set at 95 °C for 10 min, and 3% H_2_O_2_ was finally employed for blocking the activity of endogenous peroxidase. Next, the tissues were immunostained with primary antibodies (at 1:200 dilutions) at 4 °C overnight. Subsequently, the sections were incubated with corresponding secondary antibodies for 1 h at room temperature, and nuclei staining was conducted with diaminobenzidine (DAB, #D12384; Sigma Aldrich). The IHC scores of the CRC tissues for FOXA2 were quantified as follows: negative expression (<1) and positive expression (1–15). Two independent pathologists blinded to the experiments calculated the expression values for pathological scoring using ImageJ.

### Western Blot Analysis

Extraction of total protein from tissues or treated CRC cells was performed with RIPA lysis buffer (#P0013B; Beyotime Biotechnology). Then, the protein concentration was examined using a BCA protein assay kit (#P0012; Beyotime Biotechnology). An equivalent amount of protein was then loaded onto a 10% sodium dodecyl sulphate‐polyacrylamide gel (SDS‐PAGE) before transferring the proteins onto 0.45 µm PVDF membranes (Millipore). Next, 5% nonfat milk in TBST was used for the membrane blocking for 1.5 h at room temperature, followed by incubation with primary antibodies at 4 °C overnight. After washing, corresponding secondary antibodies were subjected to the membranes for 1 h of incubation at room temperature. Signals were detected using enhanced chemiluminescence (ECL, #P0018AM; Beyotime Biotechnology) following the manufacturer's instructions. The protein expression levels were quantified using ImageJ software (NIH) and normalized to the relative internal standards.

### Animal Experiments

The animal experiments were performed and approved by the Committee on Ethics in the Care and Use of Laboratory Animals of the Shandong Cancer Hospital and Institute, Shandong First Medical University, and Shandong Academy of Medical Sciences (Jinan, Shandong PR, China). All animal studies were performed in a double‐blinded manner.

Animal study‐1#: To explore the regulatory role of FOXA2 expression changes on CRC, female BALB/c nude mice (4 weeks old) were obtained from Shanghai Slack Laboratory Animals Co., Ltd. (Shanghai, China). The mice were maintained in a specific pathogen‐free (SPF) room (12 h light/dark cycle) and received an ad libitum supply of sterilized water and food. After orientation for 1 week, the mice were randomly classified into five groups: the control group (mice injected with sh‐NC‐HCT‐116 or oe‐NC‐HCT‐116 cells), the FOXA2 knockdown group (mice injected with sh‐FOXA2‐#1‐HCT‐116 or sh‐FOXA2‐#1‐HCT‐116 cells), and the FOXA2 overexpression group (mice injected with oe‐FOXA2‐HCT‐116 cells).

Animal study‐2#: To investigate the effects of OXA and FOXA2 knockdown alone or in double on CRC progression, female BALB/c nude mice (4 weeks old) were divided into four groups in a random manner, including the control group (mice only injected with HCT‐116 cells), OXA‐treated group (mice received 5 mg kg^−1^ OXA by intraperitoneal [i.p.] injection every 3 days), the FOXA2 knockdown group (mice injected with sh‐FOXA2‐HCT‐116 cells), and OXA plus FOXA2 knockdown group (mice injected with sh‐FOXA2‐HCT‐116 cells and received 5 mg/kg OXA).

The CRC xenograft mouse model was generated by subcutaneous injection of 5 × 10^6^ HCT‐116 cells in PBS (0.1 ml) into the right axilla of each animal. The tumor volume was measured every 3 days using a vernier caliper following the below equation: 0.5 × length × width^2^. After treatment for 24 days, all mice were euthanized, and the tumor weight was recorded. The collected tumors were then fixed with 4% paraformaldehyde for further detection.

Animal study‐3#: To further explore the tumorigenic role of FOXA2 in CRC in vivo, intestinal‐specific FOXA2‐knockout mice (FOXA2^f/f^; *Villin‐Cre*) with C57BL/6 background were constructed by crossing FOXA2^f/f^ conditional knockout mice with intestinal‐specific *Villin‐Cre* mice. *Villin‐Cre* mice (#004586) were purchased from the Jackson Laboratories (Bar Harbor, ME, USA), and FOXA2^flox/flox^ mice were generated as previously described.^[^ ^29^ ^]^ FOXA2^f/f^ mice were crossed with mice that expressed Cre recombinase under the control of *Villin* promoter to abrogate FOXA2 expression in an intestinal epithelial cell (IEC)‐specific manner for at least two generations. The mice were genotyped using PCR analysis followed by sequencing, and the deletion of FOXA2 protein was verified by western blot. Female mice aged at 6–8 weeks were used for experiments unless indicated otherwise. FOXA2^f/f^; *Villin‐Cre* mice were randomly separated into each group for further experiments. All investigators were blinded to the group allocation during the animal experiment. Mice with CAC were induced through AOM and DSS treatment as previously described.^[^ ^30^ ^]^ Briefly, mice at first received a single i.p. injection of 10 mg kg^−1^ AOM. After resting for 1 week, 2% DSS in drinking water was then subjected to mice for 7 consecutive days, followed by 2 weeks of regular drinking water. The cycle was subsequently repeated twice. Body weights of mice were monitored weekly throughout the experiment process. Mice in the control group were given the same volume of saline. All mice were sacrificed on week 11, and the colons were dissected from the ileocecal junction to the anus. The colon length was measured, followed by microscopical examination for tumor number counting. The colons were then fixed in 4% paraformaldehyde for hematoxylin and eosin (H&E), IHC, and IF analysis.

Animal study‐4#: Another CRC mouse model was constructed to further identify the influence of FOXA2 using male 3.5‐week‐old Apc^Min/+^ mice on the C57BL/6J background (Jackson Laboratory, Bar Harbor, ME, USA). Apc^Min/+^ mice can develop multiple adenomatous lesions in the small and large intestine that mimic the early steps in human colon cancers.^[^ ^31^ ^]^ Meanwhile, the wild‐type C57BL/6 mice were served as the normal control. Mice were maintained under SPF conditions. Recombinant AAV serotype 9 (AAV9) was used to regulate relatively high‐efficiency gene delivery.^[^ ^32^ ^]^ AAV9 carrying FOXA2 knockdown (AAV‐shFOXA2) and control recombinant AAV‐green fluorescence protein (GFP) vector 9 (AAV‐shCtrl) were constructed (GeneChem, Shanghai, China). For administration of AAV to Apc^Min/+^‐induced CRC mice, male 3.5‐week‐old Apc^Min/+^ mice (*n* = 6–10 per group) received a single intravenous dose of 2 × 10^11^ AAV‐shFOXA2 or AAV‐shCtrl in a volume of 150 µl by tail vein injection.^[^ ^33^ ^]^ Both the AAV‐shFOXA2 or AAV‐shCtrl groups of Apc^Min/+^ mice were sacrificed until the age of 20 weeks for further analysis. As for the survival of mice, AAV‐shFOXA2 and AAV‐shCtrl (*n* = 7 in each) groups of Apc^Min/+^ mice were monitored. Then, the microscopical examination to count tumor number was performed. The colons were stored at −80 °C for RT‐qPCR and western blot analysis or fixed in 4% paraformaldehyde for H&E and IHC staining.

### Co‐IP and Ubiquitination Assays

As for Co‐IP, cells were lysed in ice‐cold IP buffer (#P0013; Beyotime Biotechnology) containing the protease inhibitor (#P1006, Beyotime Biotechnology) and phosphatase inhibitor (#4906837001; Roche, Basel, Switzerland). After centrifuging at 12 000 g for 15 min, the lysates were harvested and incubated with the shown antibodies and Protein G Agarose beads (#P2053; Beyotime Biotechnology) at 4 °C overnight. After rinsing with cold IP buffer, we collected the immunocomplexes for immunoblotting with the shown primary antibodies and corresponding secondary antibodies. The ubiquitination analysis was conducted as previously described.^[^ ^34^ ^]^ The cells after transfection were cultured and lysed. Then, the indicated molecules were collected by IP using the same methods as demonstrated above. The ubiquitination condition of the proteins was analyzed by immunoblotting using the shown antibodies.

### GST Precipitation Analysis

Direct interaction between FOXA2 and TRIM36 was explored by the GST precipitation analysis as previously described.^[^ ^35^ ^]^ In brief, Rosetta (DE3) *Escherichia coli* cells were transformed with the vector pGEX‐4T‐1‐GST‐TRIM36 or pGEX‐4T‐1‐GST‐FOXA2 and then induced expression using isopropyl β‐D‐thiogalactopyranoside (#I5502; Merck, Darmstadt, Germany). The *E. coli* were lysed, and the extracts were incubated with glutathione‐Sepharose 4B beads (#RF1047; SEEBIO BIOTECH Co., Ltd., Shanghai, China) for 60 min at 4 °C. Next, the beads were incubated with purified His‐tagged TRIM36 or His‐tagged FOXA2 for another 4 h. Then, the proteins with interactions were eluted in an elution buffer, which was collected and subjected to western blot assay using anti‐His antibodies. Extract from *E. coli* only expressing a GST tag was served as the negative control.

### Statistical Analysis

All experiments were conducted with at least three biological replicates. Statistical data analysis was performed with GraphPad Prism (version 9.2.0, GraphPad Software, San Diego, CA, USA). All data are expressed as the mean ± standard deviation (SD). Differences between groups were examined using the two‐tailed Student's *t*‐test or one‐way analysis of variance. Relapse‐free‐survival (RFS) for CRC patients were analyzed by the Kaplan‐Meier (KM). The correlation between two variables was evaluated by Pearson's correlation analysis. The *p* < 0.05 was considered statistically significant.

## Conflict of Interest

The authors declare no conflict of interest.

## Supporting information

Supporting Information

## Data Availability

The data that support the findings of this study are available from the corresponding author upon reasonable request.
