# Supplementary material for: FOXA2 Suppression by TRIM36 Exerts Anti‐Tumor Role in Colorectal Cancer Via Inducing NRF2/GPX4‐Regulated Ferroptosis
Source: Adv Sci (Weinh). 2023 Oct 24;10(35):2304521. doi: 10.1002/advs.202304521 (PMC10724393; doi:10.1002/advs.202304521)
Supplement: Supplementary file 1 — Supporting Information [file ADVS-10-2304521-s001.pdf]

ADVANCED SCIENCE
Open Access

Supporting Information

for *Adv. Sci.*, DOI 10.1002/advs.202304521

FOXA2 Suppression by TRIM36 Exerts Anti-Tumor Role in Colorectal Cancer Via Inducing NRF2/GPX4-Regulated Ferroptosis

*Xin Liu, Chunli Yan, Chunxiao Chang, Fansong Meng, Wenjie Shen, Song Wang and Yi Zhang**

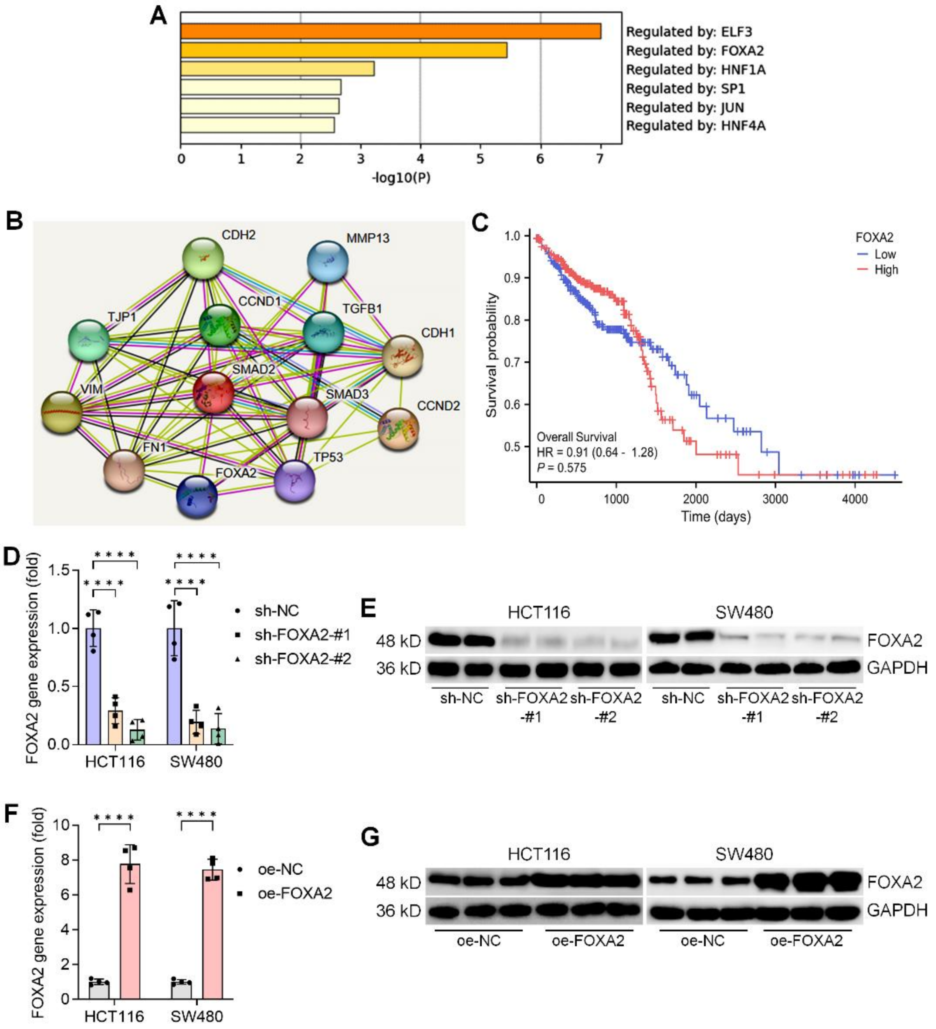

Supplementary figure 1. FOXA2 expression in CRC samples. (A) Summary of top 6 terms in TRRUST enriched for the DEGs in CRC (Metascape) from the online TCGA database. (B) STRING analysis indicating a strong interaction network between those shown proteins. (C) Overall survival rates of CRC patients by TCGA database using KM analysis. (D,E) Effect of FOXA2 knockdown (sh-FOXA2) on HCT-116 and SW480 cells by RT-qPCR and western blot assays. (F,G) RT-qPCR results of FOXA2 expression levels in CRC cells after transfection with FOXA2 over-expression plasmids (oe-FOXA2) and the negative control vector (oe-NC). n=3 or 4 in each. Data are marked as the means ± SD. ****p<0.0001.

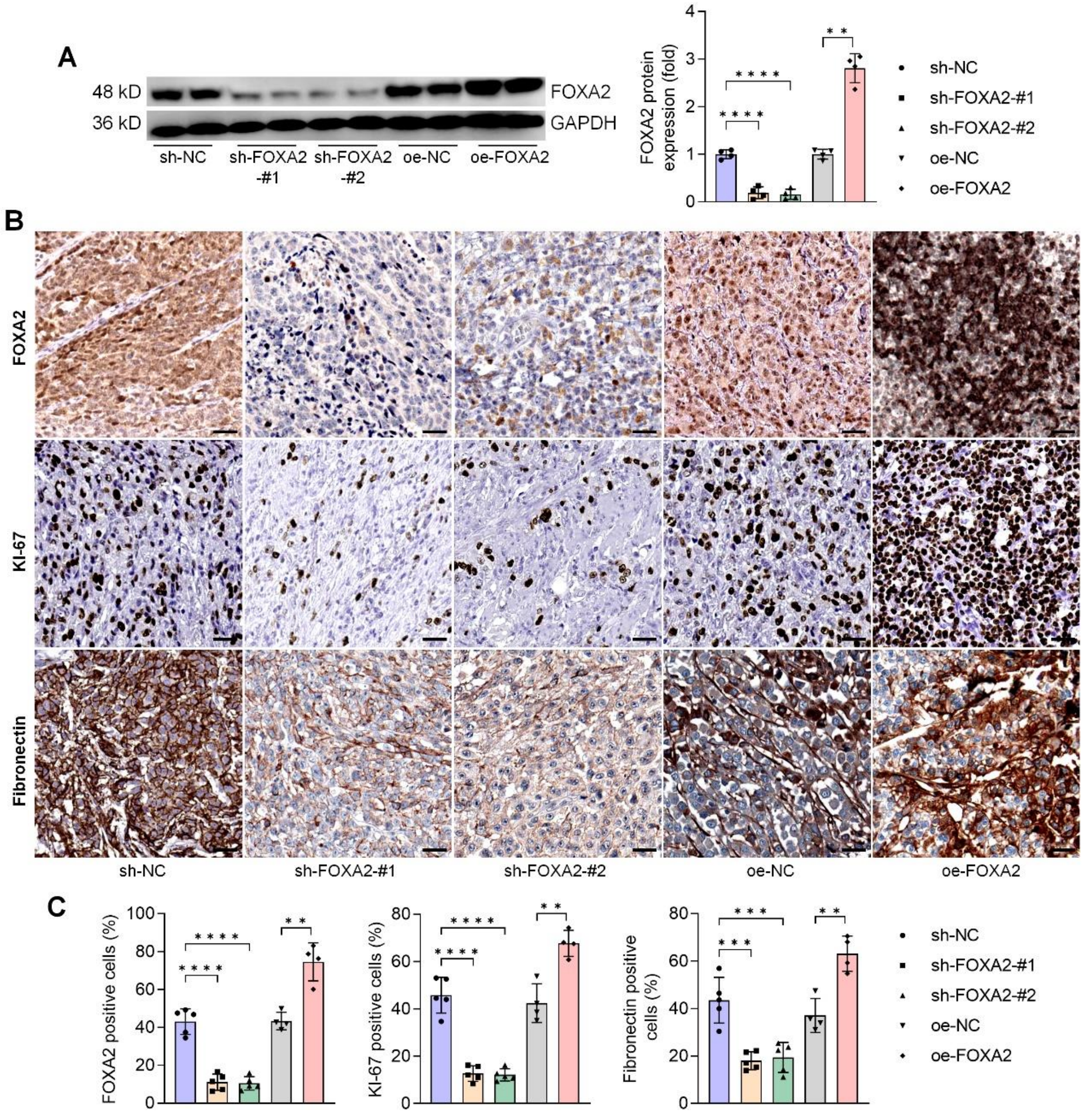

Supplementary figure 2. Effects of FOXA2 on KI-67 and Fibronectin *in vivo*. (A) Western blot analysis for FOXA2 in tumor tissues from the shown groups of mice (n=4 per group). (B,C) IHC staining for FOXA2, KI-67 and Fibronectin in tumor sections from all groups of mice (n=4 per group). Scale bar = 50 μm. Quantitative expression of these proteins was performed. Data are marked as the means ± SD. $^{**}p<0.01$, $^{***}p<0.001$, $^{****}p<0.0001$.

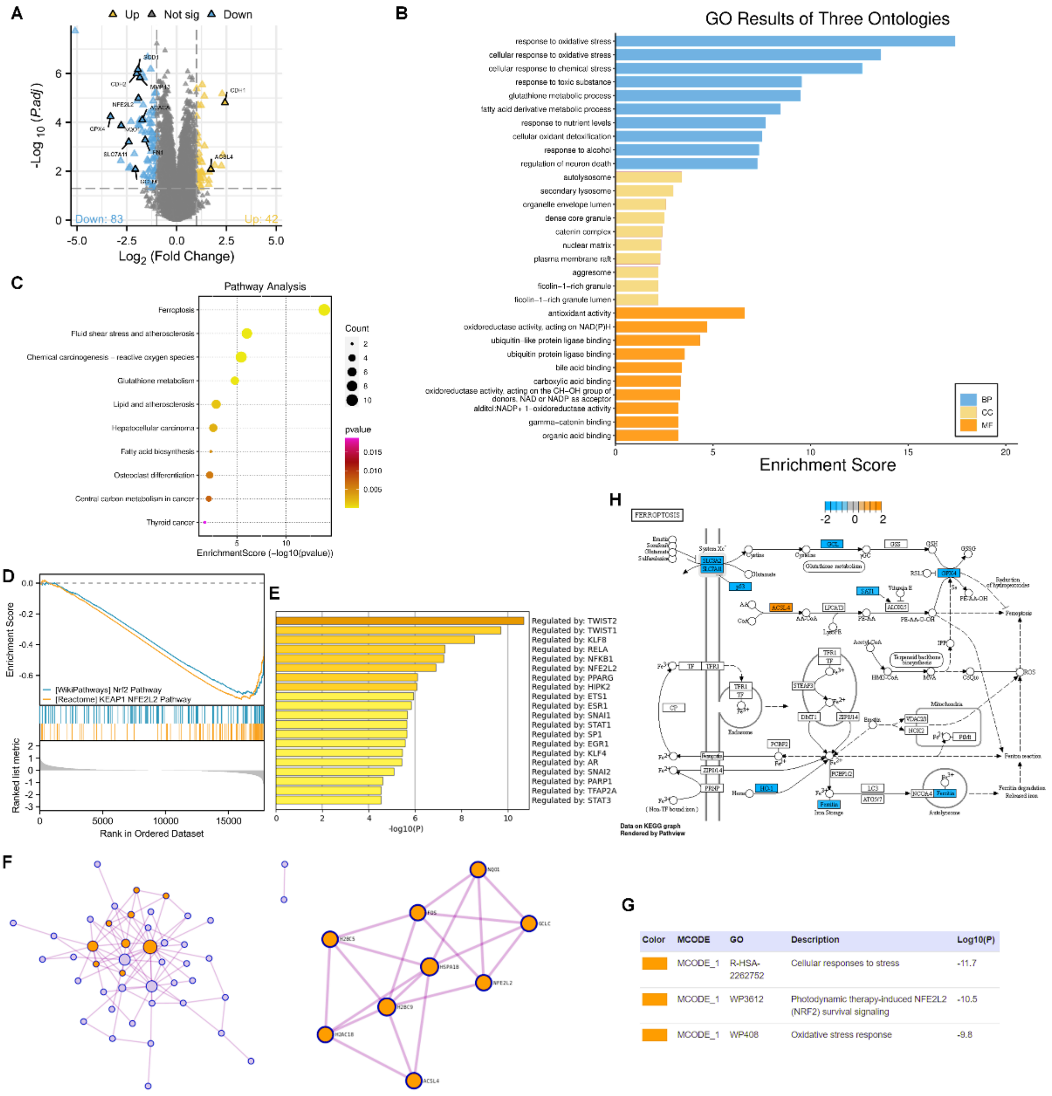

Supplementary figure 3. FOXA2 knockdown induces ferroptosis in CRC by bioinformatic analysis. (A) Volcano plot depicting genes up-regulated (yellow) or down-regulated (blue) in the HCT-116 cells with FOXA2 knockdown *vs* sh-NC (n=4 per group) via RNA sequencing assay. (B) Significantly enriched top 10 GO terms in terms of BP, CC and MF for the genes in FOXA2 knockdown HCT-116 cells. (C) KEGG enrichment analysis of DEGs between sh-FOXA2 and sh-NC HCT-116 cells based on RNA sequencing data. (D) GSEA enrichment analysis between the sh-FOXA2 *vs* sh-NC HCT-116 cells. (E) Summary of top 20 terms in TRRUST enriched for the DEGs in sh-FOXA2 HCT-116 cells *vs* sh-NC (Metascape) based on RNA sequencing analysis. (F,G) Protein-protein interaction network and MCODE components identified in the DEGs. (H) The statistically significant pathway associated with ferroptosis from the enrichment analysis result. Data are marked as the means ± SD.

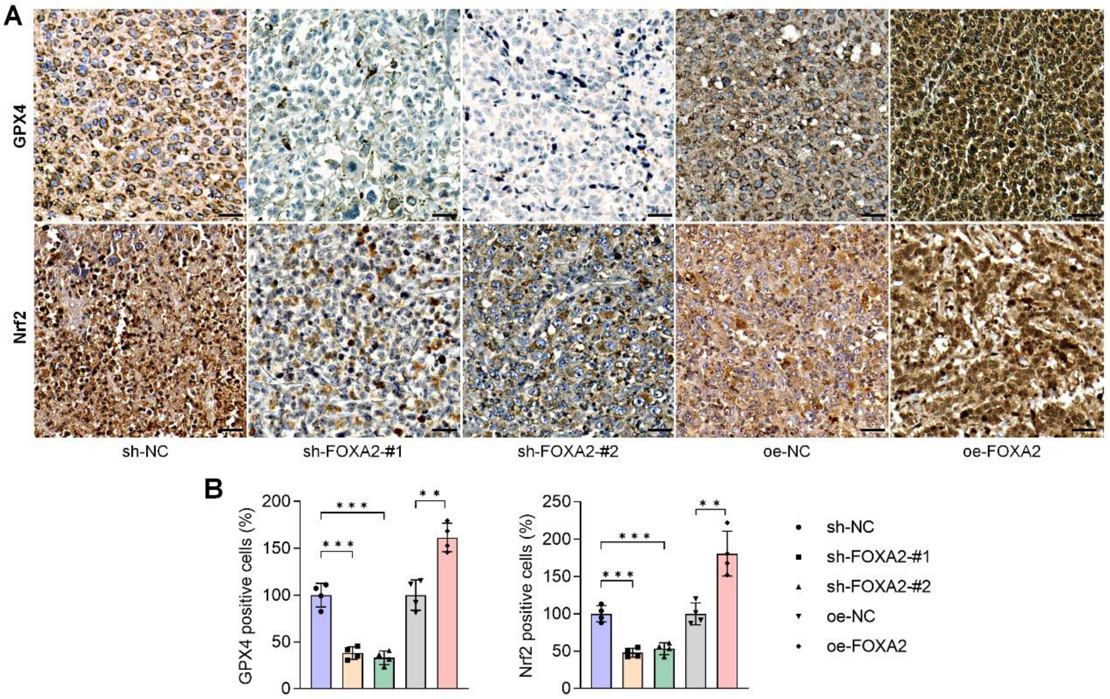

Supplementary figure 4. Influences of FOXA2 expression changes on GPX4 and Nrf2 *in vivo*. (A,B) IHC staining for GPX4 and Nrf2 in tumor sections from all groups of mice. The quantification for GPX4 and Nrf2 positive expression by IHC was performed (n=4 per group). Scale bar = 50 μm. Data are marked as the means ± SD. $^{**}p<0.01$, $^{***}p<0.001$.

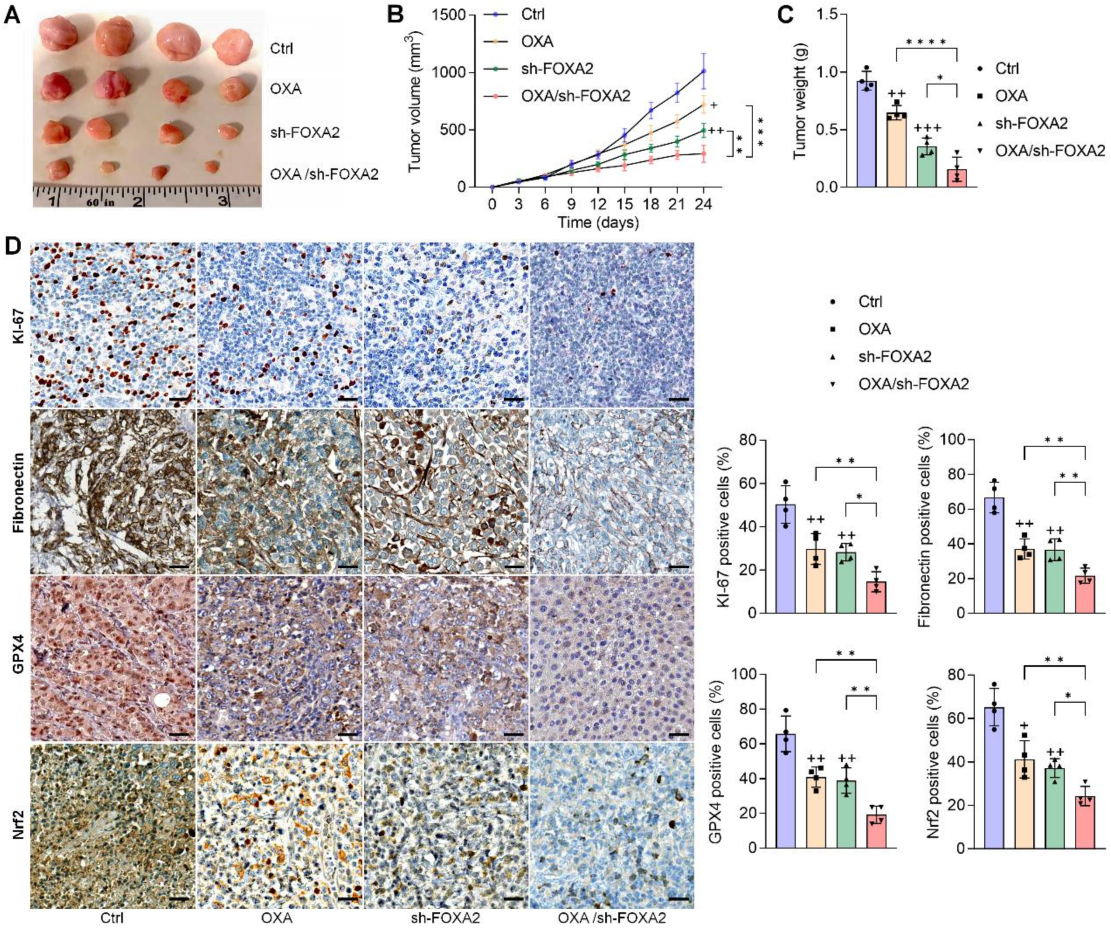

Supplementary figure 5. FOXA2 knockdown improves the anti-tumor effect of OXA on xenograft mouse model of CRC. (A) Tumor samples were displayed. (B) Tumor volume was measured. (C) Tumor weights were recorded. (D) IHC staining for KI-67, Fibronectin, GPX4 and Nrf2 was performed, and the positive expression of these proteins was quantified (n=4 per group). Scale bar = 50 μm. Data are marked as the means ± SD. $^{+}p<0.05$, $^{++}p<0.01$ versus the Ctrl group; $^{*}p<0.05$, $^{**}p<0.01$, $^{***}p<0.001$, $^{****}p<0.0001$.

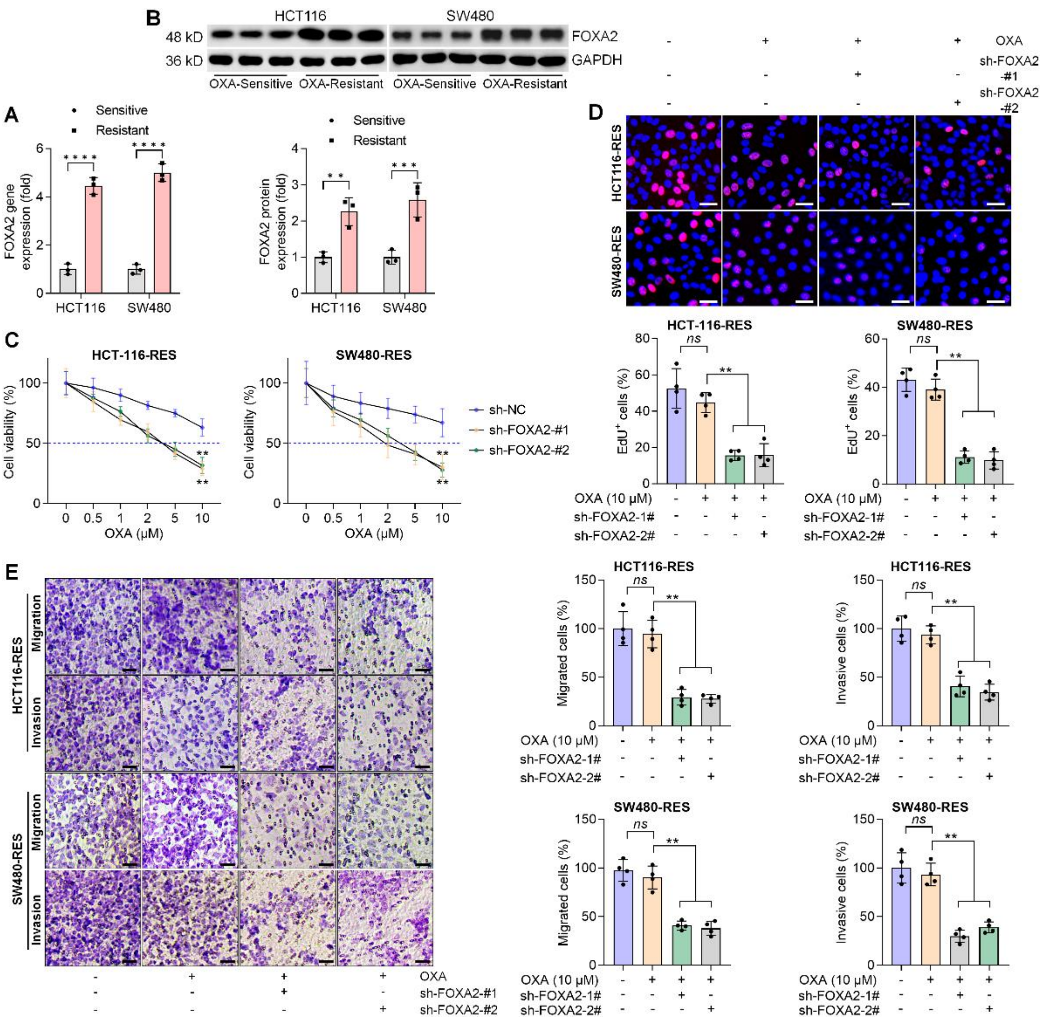

Supplementary figure 6. FOXA2 knockdown suppresses the proliferation, migration and invasion of chemoresistant CRC cells. (A) RT-qPCR and (B) western blot analysis for FOXA2 gene and protein expression levels in HCT-116 and SW480 cells with drug resistance (n=3 per group). (C) CCK-8, (D) EdU and (E) transwell analysis for the proliferation, migration and invasion of drug-resistant HCT-116 and SW480 cells (n=4 per group). Scale bar = 50 μm. Data are marked as the means ± SD. **p<0.01; *ns*, no significant difference.

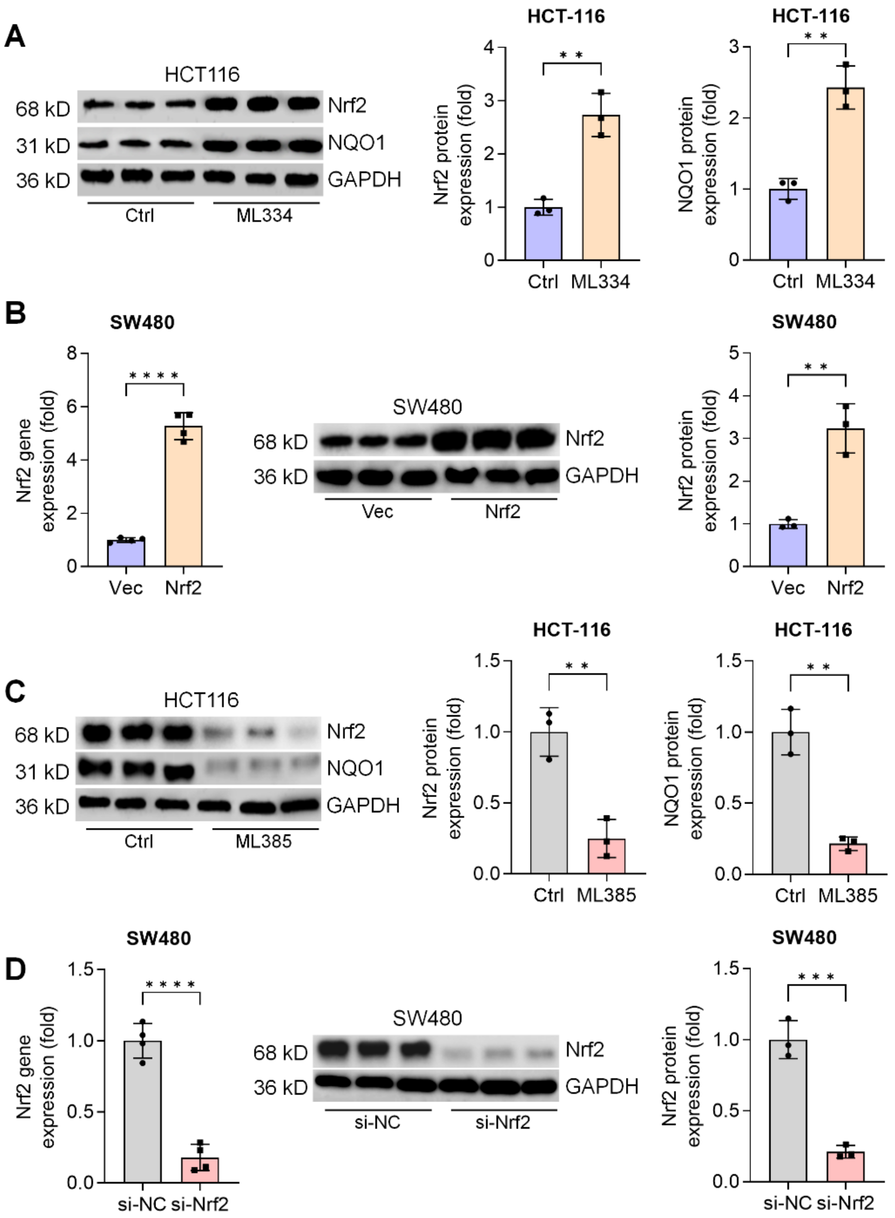

Supplementary figure 7. Nrf2 signaling calculation in CRC cells. HCT-116 cells were incubated with Nrf2 activator (ML334, 20 μM) or inhibitor (ML385, 5 μM) for 24 h. SW480 cells were transfected with Nrf2 plasmids or si-Nrf2. Then, all HCT-116 and SW480 cells were harvested for studies as follows. (A) Western blot analysis for Nrf2 and NQO1 protein levels in HCT-116 cells with ML334 exposure. (B) RT-qPCR (left) and western blot analysis (right) for Nrf2 in SW480 cells with Nrf2 over-expression. (C) Western blot analysis for Nrf2 and NQO1 protein levels in HCT-116 cells with ML385 exposure. (D) RT-qPCR (left) and western blot analysis (right) for Nrf2 in SW480 cells transfected with si-Nrf2 or si-NC. Data are marked as the means ± SD (n=3 or 4 per group). $^{**}p<0.01$, $^{***}p<0.001$, $^{****}p<0.0001$.

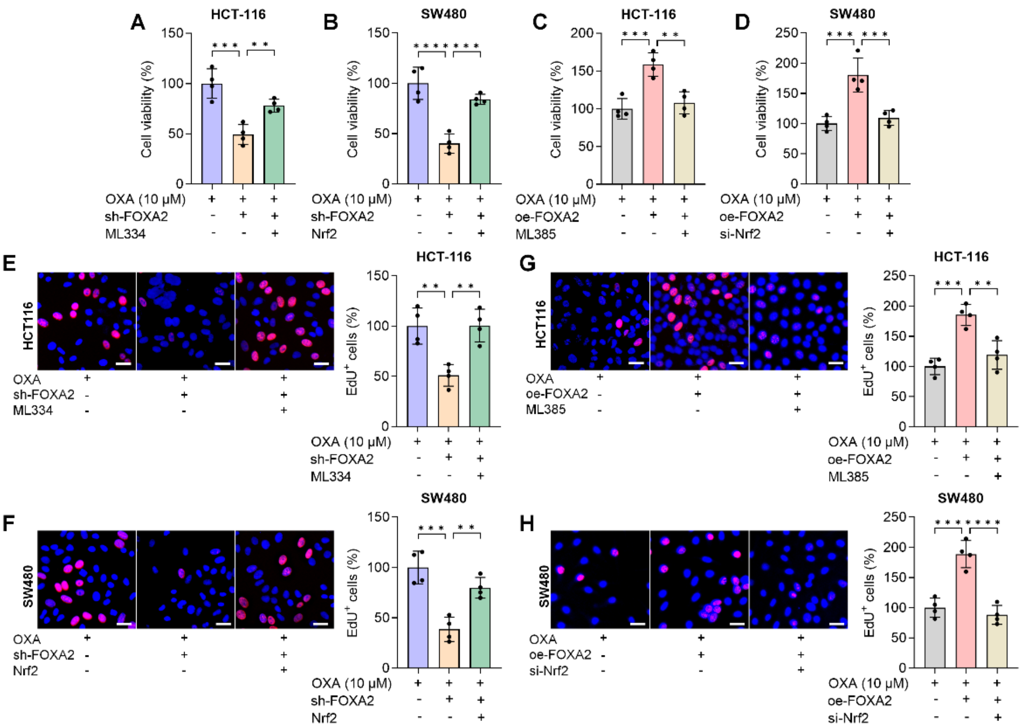

Supplementary figure 8. FOXA2 knockdown CRC cells are sensitive to OXA-reduced cell proliferation via the Nrf2 activation. HCT-116 cells with FOXA2 knockdown or over-expression were incubated with OXA (10 μM) alone or combination with Nrf2 activator (ML334, 20 μM) or inhibitor (ML385, 5 μM) for an additional 24 h. SW480 cells co-transfected with sh-FOXA2 and Nrf2 plasmids, or oe-FOXA2 and si-Nrf2 were exposed to OXA (10 μM) treatment for another 24 h. Then, all HCT-116 and SW480 cells were harvested for studies as follows. (A-D) Cell viability was measured by CCK-8 analysis. (E-H) EdU staining for cell proliferative capacity calculation. Scale bar = 50 μm. Data are marked as the means ± SD (n=4 per group). $^{**}p<0.01$, $^{***}p<0.001$, $^{****}p<0.0001$.

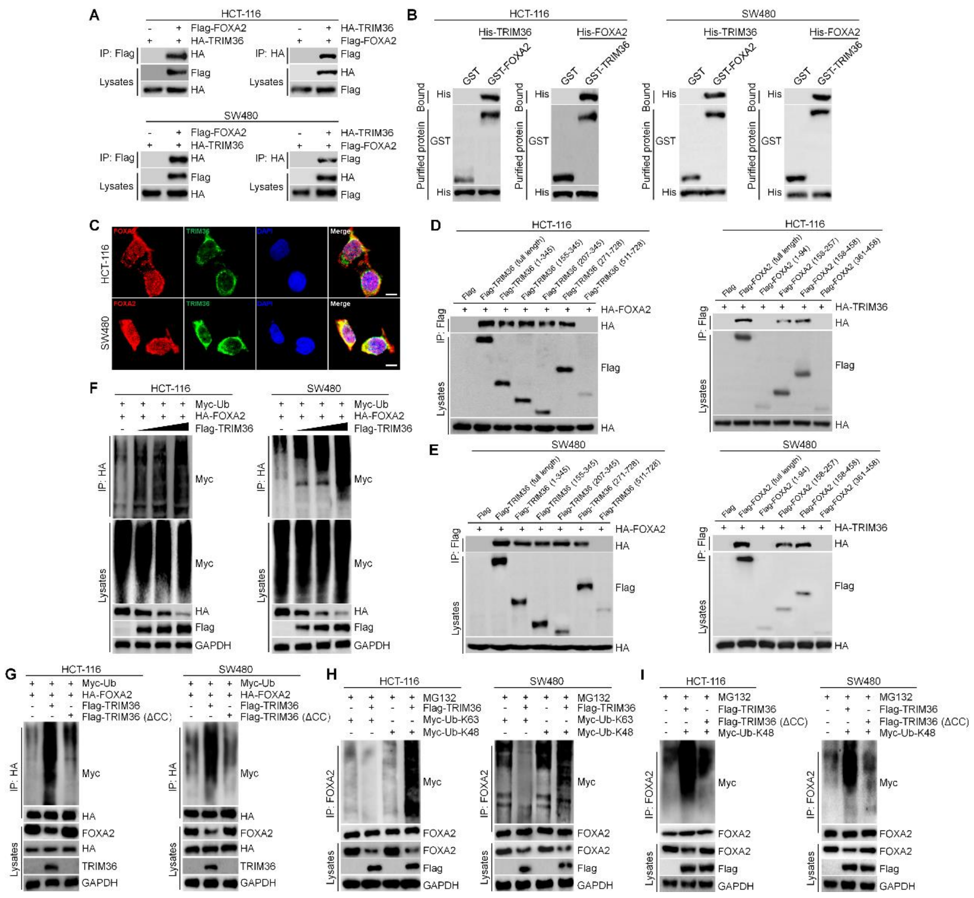

Supplementary figure 9. TRIM36 interacts with FOXA2 and induces its K48-linked polyubiquitination in colon cancer cells. (A) Co-IP analysis of HCT-116 and SW480 cells transfected with Flag-tagged FOXA2 and HA-tagged TRIM36. Anti-Flag and anti-HA antibodies were used for western blot assay. (B) GST precipitation indicating the direct interaction of TRIM36 with FOXA2 using purified GST-FOXA2 and His-tagged TRIM36 (left) or purified GST-TRIM36 and His-FOXA2 (right) by western blot analysis in HCT-116 and SW480 cells. GST was defined as a control. (C) IF images of colon cancer cells co-transfected with 24 h of Flag-tagged FOXA2 (red) and HA-tagged TRIM36 (green). Scale bar = 15 μm. (D,E) Schematic indicating full-length and truncated TRIM36 and FOXA2 with representative Co-IP assays for the mapping analysis of the domains responsible for the TRIM36/FOXA2 interaction in HCT-116 and SW480 cells. (F) Lysates of colon cancer cells transfected with plasmids expressing HA-FOXA2, Myc-Ub and increasing amounts of Flag-TIRM36 were immunoprecipitated with anti-HA beads and immunoblotted using an anti-Myc antibody. (G) Western blots showing FOXA2 ubiquitination in HCT-116 and SW480 cells transfected with the displayed plasmids in different combinations. ΔCC, deletion of the CC domain. (H) Western blot analysis indicating K48-linked ubiquitination of FOXA2 in HCT-116 and SW480 cells co-transfected with Flag-TRIM36 and Myc-K48-Ub or Myc-K63-Ub as shown. (I) Immunoblotting analysis indicating K48 ubiquitination of FOXA2 in HCT-116 and SW480 cells after co-transfection with Flag-TRIM36, Flag-TRIM36 (ΔCC) and Myc-K48-Ub. All results were representative of three independent experiments.

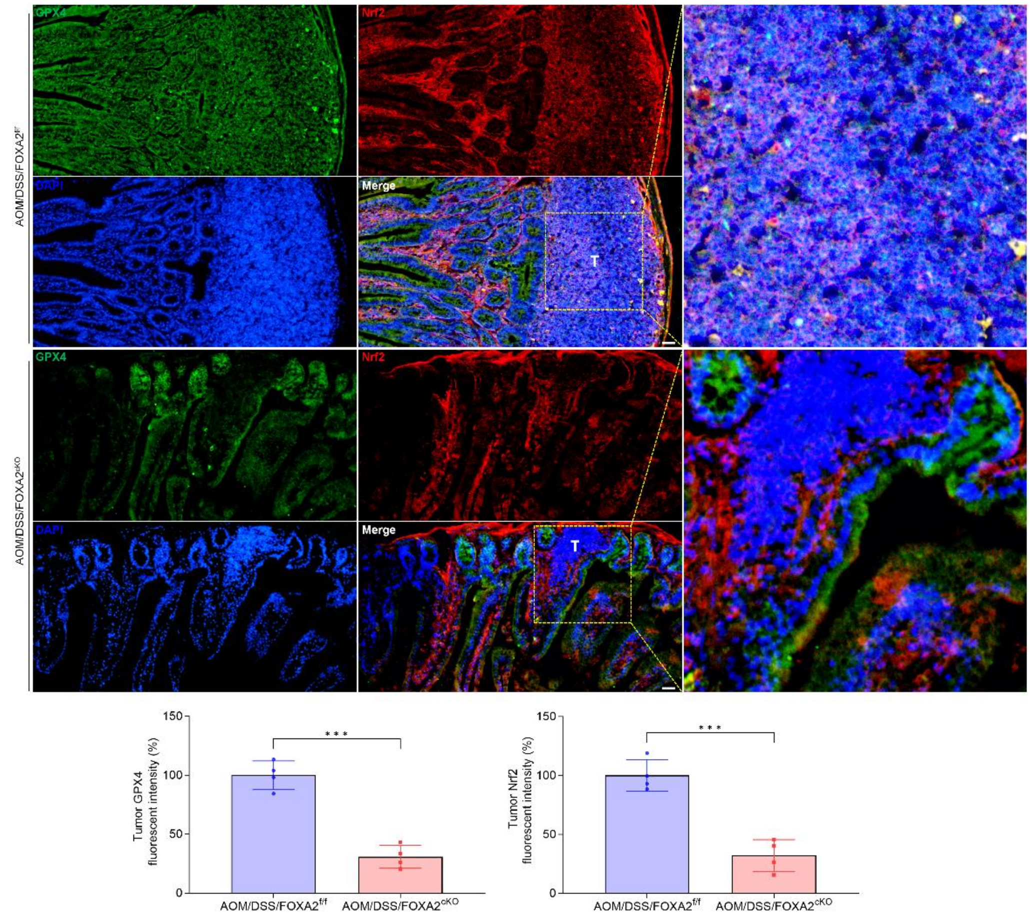

Supplementary figure 10. Calculation for GPX4 and Nrf2 in colonic tissues from AOM/DSS-treated mice bearing tumor. IF staining was performed to examine GPX4 and Nrf2 expression levels in tumor (T) areas of AOM/DSS-treated mice with or without conditional FOXA2 knockout in IECs (n=4 in each). Scale bar = 50 μm. Data are marked as the means ± SD. $^{***}p<0.001$.

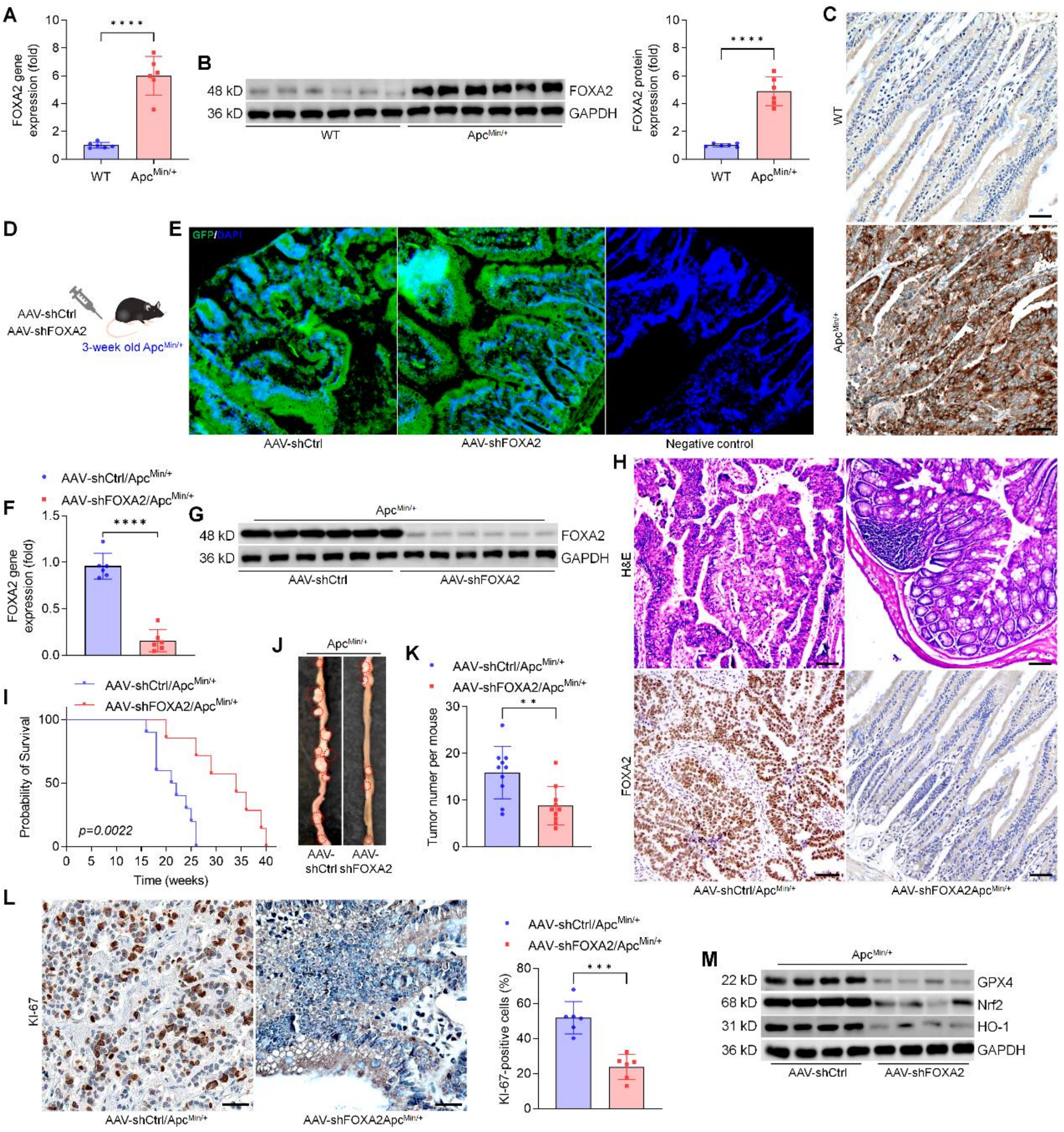

Supplementary figure 11. FOXA2 ablation in $Apc^{Min/+}$ ameliorates spontaneous intestinal tumor burden. (A) RT-qPCR, (B) western blot and (C) IHC staining assays for FOXA2 expression levels in colon tissues of $Apc^{Min/+}$ and the wild type (WT) mice (n=6 for each group). (D,E) Schematic illustration of the animal protocols for injection of shFOXA2-conjugated AAV into 3.5-week-old male $Apc^{Min/+}$ mice. Delivery of the virus was identified by GFP expression in the intestine with IF staining analysis. (F) RT-qPCR and (G) western blot analysis for FOXA2 in colon tissues of AAV-shFOXA2/$Apc^{Min/+}$ and AAV-shCtrl/$Apc^{Min/+}$ mice (n=6 in each). (H) H&E (up) and IHC staining of FOXA2 (down) in colon tissues from AAV-shFOXA2/$Apc^{Min/+}$ and AAV-shCtrl/$Apc^{Min/+}$ mice (n=4 in each). (I) Survival rates of mice were measured (n=7 per group). (J) Representative gross images of colons. (K) Tumor number on colon tissues were counted (n=10 in each). (L) IHC staining for KI-67 positive cells in colon tissues and quantification for its expression were shown (n=6 in each). (M) Western blot analysis for GPX4, Nrf2 and HO-1 expression levels in colon tissues (n=4 in each). Data are marked as the means ± SD. $^{**}p<0.01$, $^{***}p<0.001$, $^{****}p<0.0001$.

Supplementary table 1. The relationship between FOXA2 expression and clinicopathologic features of CRC patients in our cohort.

Variables	No. of cases (%)	FOXA2 expression		*P* value
		Low	High	
All	60 (100%)	30	30	
Age				
<60	27 (45.0%)	16	11	0.425
>=60	33 (55.0%)	14	19	
Gender				
Female	36 (60.0%)	17	19	0.579
Male	24 (40.0%)	13	11	
Size				
<5 cm	38 (63.3%)	23	15	0.021
>=5 cm	22 (36.7%)	7	15	
Lymph node metastasis				
No	34 (56.7%)	24	10	0.103
Yes	26 (43.3%)	6	20	
Distant metastasis				
No	47 (78.3%)	26	21	0.147
Yes	13 (21.7%)	4	9	
Clinical stage				
I + II	21 (35.0%)	14	7	0.403
III + IV	39 (65.0%)	16	23	
Differentiation				
Well and Moderate	28 (46.7%)	12	16	0.069
Poor and others	32 (53.3%)	18	14	
Clinical stage				
I + II	44 (73.3%)	24	20	0.042
III + IV	16 (26.7%)	6	10	

Supplementary table 2. Primer sequences for RT-qPCR.

**Primer name**	**Sequence 5’---3’**
FOXA2 FW	TGGAGCAGCTACTATGCAG
FOXA2 RW	CGTGTTCATGCCGTTCATC
Nrf2 FW	ATAGCTGAGCCCAGTATC
Nrf2 RW	CATGCACGTGAGTGCTCT
NQO1 FW	GGATTGGACCGAGCTGGAA
NQO1 RW	AATTGCAGTGAAGATGAAGGCAAC
G6PD FW	GAGGCCGTGTACACCAAGAT
G6PD RW	AGCAGTGGGGTGAAAATACG
SLC7A11 FW	CCTCTATTCGGACCCATTTAGT
SLC7A11 RW	CTGGGTTTCTTGTCCCATATAA
GPX4 FW	GAGGCAAGACCGAAGTAAACTAC
GPX4 RW	CCGAACTGGTTACACGGGAA
SOD1 FW	CTGAAGGCCTGCATGGATTC
SOD1 RW	CCAAGTCTCCAACATGCCTCT
GCLC FW	GAAGTGGATGTGGACACCAGATG
GCLC RW	TTGTAGTCAGGATGGTTTGCGATAA
E-Cadherin FW	CGAGAGCTACACGTTCACGG
E-Cadherin RW	GGGTGTCGAGGGAAAAATAGG
N-cadherin FW	TCAGGCGTCTGTAGAGGCTT
N-cadherin RW	ATGCACATCCTTCGATAAGACTG
Vimentin FW	GACGCCATCAACACCGAGTT
Vimentin RW	CTTTGTCGTTGGTTAGCTGGT
Fibronectin FW	GCGAGAGTGCCCCTACTACA
Fibronectin RW	GTTGGTGAATCGCAGGTCA
MMP13 FW	TGCTGCATTCTCCTTCAGGA
MMP13 RW	ATGCATCCAGGGGTCCTGGC
TGF-β1 FW	ACTACTACGCCAAGGAGGTCAC
TGF-β1 RW	GAGAGCAACACGGGTTCAGG
GAPDH FW	GCACCGTCAAGGCTGAGAAC
GAPDH RW	TGGTGAAGACGCCAGTGGA

Supplementary table 3. Detailed information on the TCGA database for OS of CRC patients.

Sample id	OS event	OS time	FOXA2
TCGA-3L-AA1B-01A-11R-A37K-07	Alive	475	5.50819727
TCGA-4N-A93T-01A-11R-A37K-07	Alive	146	5.74860882
TCGA-4T-AA8H-01A-11R-A41B-07	Alive	385	4.33763261
TCGA-5M-AAT4-01A-11R-A41B-07	Dead	49	6.46715066
TCGA-5M-AAT6-01A-11R-A41B-07	Dead	290	5.65933663
TCGA-5M-AATE-01A-11R-A41B-07	Alive	1200	4.69791835
TCGA-A6-2671-01A-01R-1410-07	Dead	1331	6.85231227
TCGA-A6-2672-01A-01R-0826-07	Alive	1419	4.8451287
TCGA-A6-2672-01B-03R-2302-07	Alive	1419	3.39833669
TCGA-A6-2674-01A-02R-0821-07	Alive	1331	6.00822467
TCGA-A6-2674-01A-02R-A278-07	Alive	1331	4.57852214
TCGA-A6-2674-01B-04R-A277-07	Alive	1331	5.34750267
TCGA-A6-2675-01A-02R-1723-07	Alive	1321	5.72094602
TCGA-A6-2676-01A-01R-0826-07	Dead	1305	5.900932
TCGA-A6-2677-01A-01R-0821-07	Dead	740	4.4910193
TCGA-A6-2677-01B-02R-A277-07	Dead	740	3.43532189
TCGA-A6-2678-01A-01R-0821-07	Alive	1286	6.0365341
TCGA-A6-2679-01A-02R-1410-07	Alive	1366	6.0374569
TCGA-A6-2680-01A-01R-1410-07	Alive	1068	5.00737044
TCGA-A6-2681-01A-01R-1410-07	Alive	1387	6.66231803
TCGA-A6-2682-01A-01R-1410-07	Dead	424	4.47283865
TCGA-A6-2683-01A-01R-0821-07	Dead	504	5.43719444
TCGA-A6-2684-01A-01R-1410-07	Alive	1127	4.56380488
TCGA-A6-2684-01A-01R-A278-07	Alive	1127	3.6107473
TCGA-A6-2684-01C-08R-A277-07	Alive	1127	4.00462723
TCGA-A6-2685-01A-01R-1410-07	Alive	1133	5.53021436
TCGA-A6-2686-01A-01R-A32Z-07	Dead	1126	5.62998602
TCGA-A6-3807-01A-01R-1022-07	Alive	1054	5.56434463
TCGA-A6-3808-01A-01R-1022-07	Alive	1014	5.40136765
TCGA-A6-3809-01A-01R-1022-07	Alive	996	4.18478808
TCGA-A6-3809-01A-01R-A278-07	Alive	996	2.52777099
TCGA-A6-3809-01B-04R-A277-07	Alive	996	2.3574957
TCGA-A6-3810-01A-01R-1022-07	Alive	1111	6.51054582
TCGA-A6-3810-01A-01R-A278-07	Alive	1111	5.16130323
TCGA-A6-3810-01B-04R-A277-07	Alive	1111	5.3374328

TCGA-A6-4105-01A-02R-1774-07	Dead	442	6.31652049
TCGA-A6-4107-01A-02R-1410-07	Alive	987	5.91318862
TCGA-A6-5656-01A-21R-1839-07	Alive	1001	5.3789624
TCGA-A6-5656-01A-21R-A278-07	Alive	1001	4.40060624
TCGA-A6-5656-01B-02R-A277-07	Alive	1001	4.42768662
TCGA-A6-5657-01A-01R-A32Z-07	Alive	962	5.61157963
TCGA-A6-5659-01A-01R-1653-07	Alive	926	6.55386886
TCGA-A6-5659-01A-01R-A278-07	Alive	926	4.85711512
TCGA-A6-5659-01B-04R-A277-07	Alive	926	4.59041639
TCGA-A6-5660-01A-01R-1653-07	Alive	888	5.78836728
TCGA-A6-5661-01A-01R-1653-07	Alive	1020	5.9731107
TCGA-A6-5661-01B-05R-2302-07	Alive	1020	2.86724414
TCGA-A6-5662-01A-01R-1653-07	Alive	718	6.65139904
TCGA-A6-5664-01A-21R-1839-07	Alive	672	6.16231277
TCGA-A6-5665-01A-01R-1653-07	Alive	671	5.72542761
TCGA-A6-5665-01B-03R-2302-07	Alive	671	4.45770591
TCGA-A6-5666-01A-01R-1653-07	Alive	995	4.74103007
TCGA-A6-5667-01A-21R-1723-07	Alive	887	6.05940503
TCGA-A6-6137-01A-11R-1774-07	Alive	824	4.68683105
TCGA-A6-6138-01A-11R-1774-07	Alive	685	6.76498965
TCGA-A6-6140-01A-11R-1774-07	Alive	734	6.32330161
TCGA-A6-6141-01A-11R-1774-07	Alive	255	6.30095102
TCGA-A6-6142-01A-11R-1774-07	Alive	763	6.66095285
TCGA-A6-6648-01A-11R-1774-07	Alive	766	5.09424029
TCGA-A6-6649-01A-11R-1774-07	Alive	735	5.30158399
TCGA-A6-6650-01A-11R-1774-07	Alive	627	6.32284572
TCGA-A6-6650-01A-11R-A278-07	Alive	627	5.85117435
TCGA-A6-6650-01B-02R-A277-07	Alive	627	5.14147775
TCGA-A6-6651-01A-21R-1839-07	Alive	662	5.63813467
TCGA-A6-6652-01A-11R-1774-07	Alive	751	6.50258998
TCGA-A6-6653-01A-11R-1774-07	Alive	742	6.71689764
TCGA-A6-6654-01A-21R-1839-07	Alive	726	5.80061004
TCGA-A6-6780-01A-11R-1839-07	Alive	612	2.11822681
TCGA-A6-6780-01A-11R-A278-07	Alive	612	1.08596845
TCGA-A6-6780-01B-04R-A277-07	Alive	612	1.02991221
TCGA-A6-6781-01A-22R-1928-07	Alive	598	5.74699256
TCGA-A6-6781-01A-22R-A278-07	Alive	598	4.97301424
TCGA-A6-6781-01B-06R-A277-07	Alive	598	3.83619683
TCGA-A6-6782-01A-11R-1839-07	Alive	617	5.82027344
TCGA-A6-A565-01A-31R-A28H-07	Dead	494	5.4663302
TCGA-A6-A566-01A-11R-A28H-07	Dead	758	3.4165155
TCGA-A6-A567-01A-31R-A28H-07	Dead	1881	4.81599035
TCGA-A6-A56B-01A-31R-A28H-07	Dead	1711	6.46572148

TCGA-A6-A5ZU-01A-11R-A28H-07	Alive	293	4.6212431
TCGA-AA-3488-01A-01R-1410-07	Dead	153	6.43993059
TCGA-AA-3489-01A-21R-1839-07	Dead	214	5.26440131
TCGA-AA-3492-01A-01R-1410-07	Dead	92	2.96781417
TCGA-AA-3494-01A-01R-1410-07	Alive	31	6.147038
TCGA-AA-3495-01A-01R-1410-07	Alive	1127	4.09206364
TCGA-AA-3496-01A-21R-1839-07	Alive	31	5.84411193
TCGA-AA-3502-01A-01R-1410-07	Alive	1065	5.86077413
TCGA-AA-3506-01A-01R-1410-07	Alive	1765	4.40020317
TCGA-AA-3509-01A-01R-1410-07	Alive	1915	5.92147442
TCGA-AA-3510-01A-01R-1410-07	Alive	1946	5.18296635
TCGA-AA-3511-01A-21R-1839-07	Alive	212	6.39103216
TCGA-AA-3514-01A-02R-0821-07	Alive	31	5.44833275
TCGA-AA-3516-01A-02R-0826-07	Dead	396	5.09801102
TCGA-AA-3517-01A-01R-0821-07	Alive	1186	5.39396528
TCGA-AA-3518-01A-02R-0826-07	Alive	31	6.33871499
TCGA-AA-3519-01A-02R-0821-07	Alive	276	4.2961637
TCGA-AA-3520-01A-01R-0821-07	Alive	731	6.45798164
TCGA-AA-3521-01A-01R-0821-07	Alive		6.39529934
TCGA-AA-3522-01A-01R-0821-07	Alive	1127	5.47750248
TCGA-AA-3524-01A-02R-0821-07	Alive	1096	3.77233965
TCGA-AA-3525-01A-02R-0826-07	Alive	245	6.13317716
TCGA-AA-3526-01A-02R-A32Z-07	Alive	580	5.24928984
TCGA-AA-3527-01A-01R-0821-07	Alive	0	4.23156297
TCGA-AA-3529-01A-02R-0821-07	Dead	0	5.83637106
TCGA-AA-3530-01A-01R-1022-07	Alive	580	6.24262537
TCGA-AA-3531-01A-01R-0821-07	Alive	1035	5.85768248
TCGA-AA-3532-01A-01R-0821-07	Alive	882	5.75961925
TCGA-AA-3534-01A-01R-0821-07	Alive	882	5.78582254
TCGA-AA-3538-01A-01R-0821-07	Alive	791	4.90932189
TCGA-AA-3542-01A-02R-1873-07	Alive	395	5.81756442
TCGA-AA-3543-01A-01R-0826-07	Alive	30	5.41553897
TCGA-AA-3544-01A-01R-1873-07	Alive	426	4.88364525
TCGA-AA-3548-01A-01R-1873-07	Alive	1034	5.73257855
TCGA-AA-3549-01A-02R-0821-07	Alive	639	6.1893047
TCGA-AA-3552-01A-01R-0821-07	Dead	396	5.07584492
TCGA-AA-3553-01A-01R-0821-07	Alive	730	4.90594773
TCGA-AA-3554-01A-01R-0826-07	Alive	546	3.98364122
TCGA-AA-3555-01A-01R-0821-07	Alive	911	7.02684542
TCGA-AA-3556-01A-01R-0821-07	Alive	700	6.57179966
TCGA-AA-3560-01A-01R-0821-07	Alive	608	4.68729589
TCGA-AA-3561-01A-01R-0821-07	Alive	424	5.40183864
TCGA-AA-3562-01A-02R-0821-07	Alive	608	5.8990886

TCGA-AA-3655-01A-02R-1723-07	Alive	1856	5.08245139
TCGA-AA-3660-01A-01R-1723-07	Alive	2375	5.34792073
TCGA-AA-3662-01A-01R-1723-07	Alive	184	4.70671841
TCGA-AA-3663-01A-01R-1723-07	Alive	212	6.54009703
TCGA-AA-3664-01A-01R-0905-07	Alive	1643	5.744928
TCGA-AA-3666-01A-02R-0905-07	Dead	61	5.65858853
TCGA-AA-3667-01A-01R-0905-07	Alive	426	5.8166509
TCGA-AA-3672-01A-01R-0905-07	Alive	28	6.01838937
TCGA-AA-3673-01A-01R-0905-07	Alive	1522	6.05916705
TCGA-AA-3675-01A-02R-0905-07	Alive	1431	5.65473626
TCGA-AA-3678-01A-01R-0905-07	Alive	1430	5.89960103
TCGA-AA-3679-01A-02R-0905-07	Alive	457	6.21608425
TCGA-AA-3680-01A-01R-0905-07	Dead	335	6.24437906
TCGA-AA-3681-01A-01R-0905-07	Alive	182	4.73927026
TCGA-AA-3684-01A-02R-0905-07	Alive	0	4.42205804
TCGA-AA-3685-01A-02R-A32Z-07	Alive	1127	5.25335398
TCGA-AA-3688-01A-01R-0905-07	Alive	578	6.14045557
TCGA-AA-3692-01A-01R-0905-07	Dead	1095	5.92017418
TCGA-AA-3693-01A-01R-0905-07	Alive	0	6.04920164
TCGA-AA-3696-01A-01R-0905-07	Dead	153	7.0851123
TCGA-AA-3697-01A-01R-1723-07	Alive	2587	5.67777601
TCGA-AA-3710-01A-01R-1022-07	Alive	821	5.67263749
TCGA-AA-3712-01A-21R-1723-07	Alive	0	5.42972307
TCGA-AA-3713-01A-21R-1723-07	Alive	579	6.43526854
TCGA-AA-3715-01A-01R-0905-07	Dead	579	5.33397108
TCGA-AA-3811-01A-01R-1022-07	Dead	306	5.38386324
TCGA-AA-3812-01A-01R-0905-07	Alive	1066	6.04976797
TCGA-AA-3814-01A-01R-0905-07	Alive	0	5.90990028
TCGA-AA-3815-01A-01R-1022-07	Alive	1005	6.07172192
TCGA-AA-3818-01A-01R-0905-07	Dead	30	6.53834881
TCGA-AA-3819-01A-01R-0905-07	Alive	761	5.53677096
TCGA-AA-3821-01A-01R-1022-07	Alive	31	7.04799745
TCGA-AA-3831-01A-01R-0905-07	Alive	547	4.47604463
TCGA-AA-3833-01A-01R-0905-07	Alive	485	5.12589489
TCGA-AA-3837-01A-01R-0905-07	Alive	1186	6.30588299
TCGA-AA-3841-01A-01R-0905-07	Alive	1124	5.49753253
TCGA-AA-3842-01A-01R-1022-07	Alive	1126	6.25967548
TCGA-AA-3844-01A-01R-1022-07	Alive	454	6.56915231
TCGA-AA-3845-01A-01R-1022-07	Dead	0	5.50959134
TCGA-AA-3846-01A-01R-1022-07	Alive	518	5.38488216
TCGA-AA-3848-01A-01R-0905-07	Dead	306	5.99820227
TCGA-AA-3850-01A-01R-1022-07	Dead	0	3.50606872
TCGA-AA-3851-01A-01R-1022-07	Alive	1006	5.15178171

TCGA-AA-3852-01A-01R-0905-07	Dead	0	6.92759784
TCGA-AA-3854-01A-01R-0905-07	Alive	1096	6.89518021
TCGA-AA-3855-01A-01R-1022-07	Alive	975	5.61499541
TCGA-AA-3856-01A-01R-0905-07	Alive	30	5.61785385
TCGA-AA-3858-01A-01R-0905-07	Alive	945	5.78520004
TCGA-AA-3860-01A-02R-0905-07	Alive	945	5.63689139
TCGA-AA-3861-01A-01R-1022-07	Alive	914	5.75293587
TCGA-AA-3862-01A-01R-1022-07	Alive	914	6.08347294
TCGA-AA-3864-01A-01R-1022-07	Alive	1612	6.27425421
TCGA-AA-3866-01A-01R-1022-07	Alive	518	5.41381069
TCGA-AA-3867-01A-01R-1022-07	Alive	731	5.78220148
TCGA-AA-3869-01A-01R-1022-07	Dead	822	5.54495825
TCGA-AA-3870-01A-01R-1022-07	Alive	912	5.72233486
TCGA-AA-3872-01A-01R-1022-07	Alive	0	5.57202864
TCGA-AA-3875-01A-01R-0905-07	Alive	549	5.05794841
TCGA-AA-3877-01A-01R-1022-07	Alive	943	5.54828248
TCGA-AA-3930-01A-01R-1022-07	Dead	61	4.65982754
TCGA-AA-3939-01A-01R-1022-07	Alive	395	4.72137813
TCGA-AA-3941-01A-01R-1022-07	Alive	730	5.74020981
TCGA-AA-3947-01A-01R-1022-07	Alive	1004	4.9939415
TCGA-AA-3949-01A-01R-1022-07	Alive	791	5.88914496
TCGA-AA-3950-01A-02R-1022-07	Alive	730	5.99915442
TCGA-AA-3952-01A-01R-1022-07	Dead	61	3.6906741
TCGA-AA-3955-01A-02R-1022-07	Alive	638	6.26578568
TCGA-AA-3956-01A-02R-1022-07	Alive	1035	5.33908036
TCGA-AA-3966-01A-01R-1113-07	Alive	61	7.15941323
TCGA-AA-3968-01A-01R-1022-07	Alive	669	5.75985623
TCGA-AA-3970-01A-01R-1022-07	Alive	1096	4.86357674
TCGA-AA-3971-01A-01R-1022-07	Alive	489	5.6085461
TCGA-AA-3972-01A-01R-1022-07	Alive	1551	5.0182024
TCGA-AA-3973-01A-01R-1022-07	Alive	397	6.92224662
TCGA-AA-3975-01A-01R-1022-07	Alive	1036	6.37685296
TCGA-AA-3976-01A-01R-1022-07	Alive	791	6.4429352
TCGA-AA-3977-01A-01R-1022-07	Alive	761	5.69369045
TCGA-AA-3979-01A-01R-1022-07	Alive	730	5.66619701
TCGA-AA-3980-01A-02R-1022-07	Alive	822	5.72297962
TCGA-AA-3982-01A-02R-1022-07	Alive	822	5.51541916
TCGA-AA-3984-01A-02R-1022-07	Alive	0	5.3878519
TCGA-AA-3986-01A-02R-1022-07	Alive	580	5.25973573
TCGA-AA-3989-01A-01R-1022-07	Dead	242	6.46236648
TCGA-AA-3994-01A-01R-1113-07	Alive	822	6.75794464
TCGA-AA-A004-01A-01R-A00A-07	Alive	424	5.25616905
TCGA-AA-A00A-01A-01R-A002-07	Alive	1157	5.57167681

TCGA-AA-A00D-01A-01R-A002-07	Alive	578	2.53876163
TCGA-AA-A00E-01A-01R-A002-07	Alive	913	6.77301125
TCGA-AA-A00F-01A-01R-A002-07	Alive	1035	6.06427715
TCGA-AA-A00J-01A-02R-A002-07	Alive	549	6.80504218
TCGA-AA-A00K-01A-02R-A002-07	Alive	549	5.2152204
TCGA-AA-A00L-01A-01R-A002-07	Alive	1157	6.17446634
TCGA-AA-A00N-01A-02R-A00A-07	Dead	122	5.41782552
TCGA-AA-A00O-01A-02R-A089-07	Alive	822	4.83581799
TCGA-AA-A00Q-01A-01R-A002-07	Alive	1278	5.98993826
TCGA-AA-A00R-01A-01R-A002-07	Alive	30	0.15445359
TCGA-AA-A00U-01A-01R-A002-07	Alive	518	6.3870747
TCGA-AA-A00W-01A-01R-A002-07	Alive	456	6.71004184
TCGA-AA-A00Z-01A-01R-A002-07	Alive	669	5.82847418
TCGA-AA-A010-01A-01R-A089-07	Alive	1064	5.46860287
TCGA-AA-A017-01A-01R-A00A-07	Alive	457	5.04290694
TCGA-AA-A01C-01A-01R-A00A-07	Alive	457	6.3244272
TCGA-AA-A01D-01A-01R-A00A-07	Dead	334	5.30800039
TCGA-AA-A01F-01A-01R-A002-07	Alive	974	6.09735164
TCGA-AA-A01G-01A-01R-A002-07	Alive	365	5.90206152
TCGA-AA-A01I-01A-02R-A089-07	Alive	943	4.97885458
TCGA-AA-A01K-01A-01R-A00A-07	Alive	943	4.7956319
TCGA-AA-A01P-01A-21R-A083-07	Dead	1158	4.86309597
TCGA-AA-A01Q-01A-01R-A002-07	Alive	31	5.52280905
TCGA-AA-A01R-01A-21R-A083-07	Alive	1065	1.06591692
TCGA-AA-A01S-01A-21R-A083-07	Alive	31	5.40485204
TCGA-AA-A01T-01A-21R-A16W-07	Alive	1005	5.88489092
TCGA-AA-A01V-01A-23R-A083-07	Alive	31	5.76092083
TCGA-AA-A01X-01A-21R-A083-07	Alive	791	5.66873184
TCGA-AA-A01Z-01A-11R-A083-07	Alive	1126	5.84154296
TCGA-AA-A022-01A-21R-A16W-07	Alive	0	4.53631405
TCGA-AA-A024-01A-02R-A00A-07	Dead	1188	5.85792876
TCGA-AA-A029-01A-01R-A00A-07	Alive	1581	4.00279252
TCGA-AA-A02E-01A-01R-A00A-07	Dead	90	5.97350337
TCGA-AA-A02F-01A-01R-A089-07	Alive	1216	6.96881192
TCGA-AA-A02H-01A-01R-A089-07	Dead	61	6.39480221
TCGA-AA-A02J-01A-01R-A00A-07	Dead	153	6.51979663
TCGA-AA-A02K-01A-03R-A32Y-07	Dead	426	5.01644729
TCGA-AA-A02O-01A-21R-A16W-07	Alive	28	6.02889399
TCGA-AA-A02R-01A-01R-A00A-07	Dead	670	5.1356662
TCGA-AA-A02W-01A-01R-A00A-07	Alive	1247	7.01079326
TCGA-AA-A02Y-01A-43R-A32Y-07	Alive	1216	6.08922906
TCGA-AA-A03F-01A-11R-A16W-07	Dead	549	4.97762338
TCGA-AA-A03J-01A-21R-A16W-07	Alive	1246	6.2580157

TCGA-AD-5900-01A-11R-1653-07	Alive	370	5.64184367
TCGA-AD-6548-01A-11R-1839-07	Alive	650	4.38820332
TCGA-AD-6888-01A-11R-1928-07	Dead	472	5.78900668
TCGA-AD-6889-01A-11R-1928-07	Dead	2532	6.22551981
TCGA-AD-6890-01A-11R-1928-07	Alive	746	4.39582372
TCGA-AD-6895-01A-11R-1928-07	Alive	763	6.43013136
TCGA-AD-6899-01A-11R-1928-07	Dead	176	4.90881771
TCGA-AD-6901-01A-11R-1928-07	Dead	682	6.74765056
TCGA-AD-6963-01A-11R-1928-07	Alive	834	5.25744442
TCGA-AD-6964-01A-11R-1928-07	Dead	331	5.96710634
TCGA-AD-6965-01A-11R-1928-07	Alive	805	6.03461631
TCGA-AD-A5EJ-01A-11R-A28H-07	Alive	0	6.6095112
TCGA-AD-A5EK-01A-11R-A28H-07	Alive	500	6.7559571
TCGA-AF-2687-01A-02R-1736-07	Alive	1427	5.72159687
TCGA-AF-2690-01A-02R-1736-07	Dead	524	5.01510055
TCGA-AF-2691-01A-01R-0821-07	Alive	1309	6.23019917
TCGA-AF-2692-01A-01R-0821-07	Alive	412	5.94636593
TCGA-AF-2693-01A-02R-1736-07	Alive	1155	5.84146497
TCGA-AF-3400-01A-01R-0821-07	Alive	1049	5.75294657
TCGA-AF-3911-01A-01R-1736-07	Alive	1148	5.25841339
TCGA-AF-3913-01A-02R-1119-07	Dead	316	6.26529624
TCGA-AF-4110-01A-02R-1736-07	Alive	912	5.38070867
TCGA-AF-5654-01A-01R-1660-07	Dead	512	6.53166841
TCGA-AF-6136-01A-11R-1830-07	Alive	741	6.26421363
TCGA-AF-6655-01A-11R-1830-07	Alive	609	6.21147951
TCGA-AF-6672-01A-11R-1830-07	Alive	748	6.93045114
TCGA-AF-A56K-01A-32R-A39D-07	Alive	2635	5.34953159
TCGA-AF-A56L-01A-31R-A39D-07	Alive	2007	5.41713014
TCGA-AF-A56N-01A-12R-A39D-07	Alive	360	6.63280912
TCGA-AG-3574-01A-01R-0821-07	Dead	1096	5.25096157
TCGA-AG-3575-01A-01R-0821-07	Alive	365	1.32083123
TCGA-AG-3578-01A-01R-0821-07	Alive	974	6.87776388
TCGA-AG-3580-01A-01R-0821-07	Alive	244	5.86195288
TCGA-AG-3581-01A-01R-0821-07	Alive	215	5.60383995
TCGA-AG-3582-01A-01R-0821-07	Dead	1096	6.62620694
TCGA-AG-3583-01A-01R-0821-07	Dead	610	5.26156842
TCGA-AG-3584-01A-01R-0821-07	Dead	730	5.60678865
TCGA-AG-3586-01A-02R-0821-07	Alive	31	5.03013299
TCGA-AG-3587-01A-01R-0821-07	Alive	1400	6.66132064
TCGA-AG-3591-01A-01R-1736-07	Alive	1035	6.2645552
TCGA-AG-3592-01A-02R-1736-07	Alive	1035	5.96937386
TCGA-AG-3593-01A-01R-0821-07	Alive	1035	5.9437057
TCGA-AG-3594-01A-02R-0821-07	Dead	61	6.89716054

TCGA-AG-3598-01A-01R-0826-07	Alive	1522	5.50179898
TCGA-AG-3599-01A-02R-0826-07	Alive	366	6.36585698
TCGA-AG-3600-01A-01R-0826-07	Alive	184	5.94581602
TCGA-AG-3601-01A-01R-0826-07	Alive	0	5.94065775
TCGA-AG-3602-01A-02R-0826-07	Alive	0	4.23463927
TCGA-AG-3605-01A-01R-0826-07	Alive	30	6.23277586
TCGA-AG-3608-01A-01R-0826-07	Alive	485	4.84933879
TCGA-AG-3609-01A-02R-0826-07	Alive	608	6.1139359
TCGA-AG-3611-01A-01R-0826-07	Alive	424	7.01785289
TCGA-AG-3612-01A-01R-0826-07	Alive	608	6.7227406
TCGA-AG-3725-01A-11R-1736-07	Alive	638	6.15180809
TCGA-AG-3726-01A-02R-0905-07	Alive	243	7.12958183
TCGA-AG-3727-01A-01R-0905-07	Alive	30	7.33351945
TCGA-AG-3728-01A-01R-0905-07	Alive	912	6.912281
TCGA-AG-3731-01A-11R-1736-07	Alive	1126	5.99865586
TCGA-AG-3732-01A-11R-1660-07	Alive	1003	7.03134667
TCGA-AG-3742-01A-11R-1660-07	Alive	30	5.61369371
TCGA-AG-3878-01A-02R-0905-07	Alive	30	6.04694281
TCGA-AG-3881-01A-01R-0905-07	Alive	579	7.15047743
TCGA-AG-3882-01A-01R-0905-07	Alive	608	6.31114523
TCGA-AG-3883-01A-02R-0905-07	Alive	31	5.85119934
TCGA-AG-3885-01A-01R-0905-07	Alive	546	6.7545348
TCGA-AG-3887-01A-01R-1119-07	Alive	1124	6.25659367
TCGA-AG-3890-01A-01R-1119-07	Alive	518	5.48794612
TCGA-AG-3892-01A-01R-1119-07	Alive	396	4.75852172
TCGA-AG-3893-01A-01R-1119-07	Alive	1065	5.77841887
TCGA-AG-3894-01A-01R-1119-07	Alive	426	6.68619795
TCGA-AG-3896-01A-01R-1119-07	Alive	31	6.78641863
TCGA-AG-3898-01A-01R-1119-07	Alive	1461	4.83295076
TCGA-AG-3901-01A-01R-1119-07	Alive	761	6.01072166
TCGA-AG-3902-01A-01R-A32Z-07	Alive	974	4.9624426
TCGA-AG-3909-01A-01R-1119-07	Alive	608	6.94866976
TCGA-AG-3999-01A-01R-1119-07	Alive	853	6.13441194
TCGA-AG-4001-01A-02R-1119-07	Alive	1096	6.76452132
TCGA-AG-4005-01A-01R-1119-07	Alive	427	6.51371654
TCGA-AG-4007-01A-01R-1119-07	Alive	31	6.51533084
TCGA-AG-4008-01A-01R-1119-07	Alive	518	5.48245465
TCGA-AG-4015-01A-01R-1119-07	Alive	0	6.19405947
TCGA-AG-4021-01A-01R-1736-07	Dead	121	4.49969284
TCGA-AG-4022-01A-01R-1736-07	Alive	1400	5.46617682
TCGA-AG-A002-01A-01R-A002-07	Alive	638	4.89698382
TCGA-AG-A008-01A-01R-A002-07	Alive	424	6.27810187
TCGA-AG-A00C-01A-01R-A002-07	Alive	183	5.77386977

TCGA-AG-A00H-01A-01R-A00A-07	Alive	790	5.97122164
TCGA-AG-A00Y-01A-02R-A002-07	Alive	700	7.03433569
TCGA-AG-A011-01A-01R-A002-07	Alive	1126	4.72694563
TCGA-AG-A014-01A-02R-A002-07	Alive	485	2.98924802
TCGA-AG-A015-01A-01R-A002-07	Alive	1096	6.92609077
TCGA-AG-A016-01A-01R-A002-07	Alive	276	6.40551932
TCGA-AG-A01J-01A-01R-A00A-07	Alive	31	5.49629936
TCGA-AG-A01L-01A-01R-A002-07	Alive	0	6.78983096
TCGA-AG-A01N-01A-01R-A00A-07	Alive	943	6.21206734
TCGA-AG-A01W-01A-21R-A083-07	Alive	0	5.8228321
TCGA-AG-A01Y-01A-41R-A083-07	Alive	0	5.69587414
TCGA-AG-A020-01A-21R-A083-07	Alive	31	6.6800247
TCGA-AG-A023-01A-01R-A00A-07	Dead	1581	5.44573994
TCGA-AG-A025-01A-01R-A00A-07	Alive	1520	4.79551242
TCGA-AG-A026-01A-01R-A00A-07	Dead	59	6.13434828
TCGA-AG-A02G-01A-01R-A00A-07	Dead	1185	7.27878582
TCGA-AG-A02N-01A-11R-A083-07	Alive	1885	6.16190783
TCGA-AG-A02X-01A-01R-A00A-07	Alive	1247	8.23825616
TCGA-AG-A032-01A-01R-A00A-07	Alive	1157	6.57653272
TCGA-AG-A036-01A-12R-A083-07	Alive	3562	5.83478452
TCGA-AH-6544-01A-11R-1830-07	Alive	1173	5.62135154
TCGA-AH-6547-01A-11R-1830-07	Dead	76	5.43369059
TCGA-AH-6549-01A-11R-1830-07	Alive	532	6.28567778
TCGA-AH-6643-01A-11R-1830-07	Dead	1314	6.11836596
TCGA-AH-6644-01A-21R-1830-07	Alive	838	6.58880157
TCGA-AH-6897-01A-11R-1928-07	Alive	804	5.90966511
TCGA-AH-6903-01A-11R-1928-07	Alive	592	5.64357628
TCGA-AM-5820-01A-01R-1653-07	Alive	14	4.35594655
TCGA-AM-5821-01A-01R-1653-07	Alive	28	5.56668731
TCGA-AU-3779-01A-01R-1723-07	Alive	441	6.14219489
TCGA-AU-6004-01A-11R-1723-07	Alive	824	4.06779358
TCGA-AY-4070-01A-01R-1113-07	Dead	496	5.91126734
TCGA-AY-4071-01A-01R-1113-07	Dead	29	5.76397983
TCGA-AY-5543-01A-01R-1653-07	Alive	1004	6.06130098
TCGA-AY-6196-01A-11R-1723-07	Alive	6	5.68757025
TCGA-AY-6197-01A-11R-1723-07	Alive	652	3.08812463
TCGA-AY-6386-01A-21R-1723-07	Alive	542	5.09304882
TCGA-AY-A54L-01A-11R-A28H-07	Alive	525	4.90841904
TCGA-AY-A69D-01A-11R-A37K-07	Alive	543	6.51172894
TCGA-AY-A71X-01A-12R-A37K-07	Alive	588	6.33379581
TCGA-AY-A8YK-01A-11R-A41B-07	Alive	573	5.32311422
TCGA-AZ-4308-01A-01R-1410-07	Alive	3324	5.31670692
TCGA-AZ-4313-01A-01R-1410-07	Alive	2310	6.36014828

TCGA-AZ-4315-01A-01R-1410-07	Alive	1776	3.72339473
TCGA-AZ-4323-01A-21R-1839-07	Dead	43	5.59737921
TCGA-AZ-4614-01A-01R-1410-07	Dead	172	6.23811044
TCGA-AZ-4615-01A-01R-1410-07	Alive	1002	5.3151097
TCGA-AZ-4616-01A-21R-1839-07	Dead	156	4.70767387
TCGA-AZ-4682-01B-01R-A32Z-07	Dead	680	4.7540644
TCGA-AZ-4684-01A-01R-1410-07	Alive	1977	4.40411972
TCGA-AZ-5403-01A-01R-1653-07	Dead	1910	5.32142667
TCGA-AZ-5407-01A-01R-1723-07	Alive	2683	4.73558175
TCGA-AZ-6598-01A-11R-1774-07	Dead	1503	7.6216314
TCGA-AZ-6599-01A-11R-1774-07	Dead	206	8.22480482
TCGA-AZ-6600-01A-11R-1774-07	Dead	368	5.51047778
TCGA-AZ-6601-01A-11R-1774-07	Dead	3042	5.29804973
TCGA-AZ-6603-01A-11R-1839-07	Dead	899	6.50775659
TCGA-AZ-6605-01A-11R-1839-07	Dead	159	5.03823862
TCGA-AZ-6606-01A-11R-1839-07	Dead	357	6.02120209
TCGA-AZ-6607-01A-11R-1839-07	Dead	97	4.62646834
TCGA-AZ-6608-01A-11R-1839-07	Dead	59	6.47535893
TCGA-BM-6198-01A-11R-1736-07	Alive	646	5.99776886
TCGA-CA-5254-01A-21R-1839-07	Alive	386	5.19696107
TCGA-CA-5255-01A-11R-1839-07	Alive	376	5.56575862
TCGA-CA-5256-01A-01R-1410-07	Alive	379	5.08373247
TCGA-CA-5796-01A-01R-1653-07	Alive	377	6.04132623
TCGA-CA-5797-01A-01R-1653-07	Alive	383	6.32735324
TCGA-CA-6715-01A-21R-1839-07	Alive	383	6.33615304
TCGA-CA-6716-01A-11R-1839-07	Alive	371	7.17855431
TCGA-CA-6717-01A-11R-1839-07	Alive	388	4.10060768
TCGA-CA-6718-01A-11R-1839-07	Dead	306	4.61055834
TCGA-CA-6719-01A-11R-1839-07	Alive	435	6.86137231
TCGA-CI-6619-01B-11R-1830-07	Alive	184	4.79356299
TCGA-CI-6620-01A-11R-1830-07	Alive	1009	6.1999687
TCGA-CI-6621-01A-11R-1830-07	Alive	419	6.24001087
TCGA-CI-6622-01A-11R-1830-07	Alive	1362	5.45788973
TCGA-CI-6623-01B-11R-1830-07	Alive	1443	6.09809737
TCGA-CI-6624-01C-11R-1830-07	Alive	1466	5.54176262
TCGA-CK-4947-01B-11R-1653-07	Alive	534	5.8869549
TCGA-CK-4948-01B-11R-1653-07	Alive	4502	4.61421512
TCGA-CK-4950-01A-01R-1723-07	Alive	2599	5.35802696
TCGA-CK-4951-01A-01R-1410-07	Dead	2134	4.78350904
TCGA-CK-4952-01A-01R-1723-07	Alive	475	7.44550987
TCGA-CK-5912-01A-11R-1653-07	Dead	1493	6.63343266
TCGA-CK-5913-01A-11R-1653-07	Alive	1561	6.00257201
TCGA-CK-5914-01A-11R-1653-07	Alive	669	6.10105962

TCGA-CK-5915-01A-11R-1653-07	Alive	0	6.25094452
TCGA-CK-5916-01A-11R-1653-07	Dead	643	5.16104922
TCGA-CK-6746-01A-11R-1839-07	Alive	0	7.57526316
TCGA-CK-6747-01A-11R-1839-07	Alive	2523	4.79108473
TCGA-CK-6748-01A-11R-1839-07	Alive	61	7.16785465
TCGA-CK-6751-01A-11R-1839-07	Alive	3780	4.56387808
TCGA-CL-4957-01A-01R-1736-07	Alive	425	5.98798475
TCGA-CL-5917-01A-11R-1660-07	Alive	2376	6.07488644
TCGA-CL-5918-01A-11R-1660-07	Alive	218	5.50795634
TCGA-CM-4743-01A-01R-1723-07	Alive	701	4.96542414
TCGA-CM-4744-01A-01R-A32Z-07	Alive	609	3.36819563
TCGA-CM-4746-01A-01R-1410-07	Alive	1126	6.36397527
TCGA-CM-4747-01A-01R-1410-07	Alive	761	6.63641726
TCGA-CM-4748-01A-01R-1410-07	Alive	792	6.65152964
TCGA-CM-4750-01A-01R-1410-07	Alive	244	5.36494706
TCGA-CM-4751-01A-02R-1839-07	Alive	822	4.5438794
TCGA-CM-4752-01A-01R-1410-07	Alive	396	5.5158039
TCGA-CM-5341-01A-01R-1410-07	Alive	884	4.6631795
TCGA-CM-5344-01A-21R-1723-07	Alive	670	6.75971512
TCGA-CM-5348-01A-21R-1723-07	Alive	699	5.25427657
TCGA-CM-5349-01A-21R-1723-07	Alive	915	6.30137181
TCGA-CM-5860-01A-01R-1653-07	Alive	974	6.04728972
TCGA-CM-5861-01A-01R-1653-07	Alive	457	6.58492042
TCGA-CM-5862-01A-01R-1653-07	Dead	153	5.57096378
TCGA-CM-5863-01A-21R-1839-07	Alive	457	6.15298041
TCGA-CM-5864-01A-01R-1653-07	Alive	457	5.28830319
TCGA-CM-5868-01A-01R-1653-07	Alive	518	6.42668408
TCGA-CM-6161-01A-11R-1653-07	Alive	457	6.54984934
TCGA-CM-6162-01A-11R-1653-07	Alive	365	5.51979663
TCGA-CM-6163-01A-11R-1653-07	Alive	427	5.04131746
TCGA-CM-6164-01A-11R-1653-07	Alive	883	6.18839499
TCGA-CM-6165-01A-11R-1653-07	Alive	488	6.02919224
TCGA-CM-6166-01A-11R-1653-07	Alive	669	6.68605224
TCGA-CM-6167-01A-11R-1653-07	Alive	456	4.63768559
TCGA-CM-6168-01A-11R-1653-07	Alive	395	4.55297336
TCGA-CM-6169-01A-11R-1653-07	Alive	396	4.5797172
TCGA-CM-6170-01A-11R-1653-07	Alive	457	6.29613616
TCGA-CM-6171-01A-11R-1653-07	Alive	427	7.56471959
TCGA-CM-6172-01A-11R-1653-07	Alive	335	4.8788087
TCGA-CM-6674-01A-11R-1839-07	Alive	394	6.17831428
TCGA-CM-6675-01A-11R-1839-07	Alive	397	5.08425139
TCGA-CM-6676-01A-11R-1839-07	Alive	337	5.80044696
TCGA-CM-6677-01A-11R-1839-07	Alive	337	5.17836408

TCGA-CM-6678-01A-11R-1839-07	Alive	335	6.36970377
TCGA-CM-6679-01A-11R-1839-07	Alive	306	6.52724397
TCGA-CM-6680-01A-11R-1839-07	Alive	366	6.2157873
TCGA-D5-5537-01A-21R-1928-07	Dead	1381	6.1310585
TCGA-D5-5538-01A-01R-1653-07	Dead	1661	5.50996814
TCGA-D5-5539-01A-01R-1653-07	Alive	596	6.62717052
TCGA-D5-5540-01A-01R-1653-07	Alive	1706	5.80200964
TCGA-D5-5541-01A-01R-1653-07	Alive	1701	5.70475303
TCGA-D5-6529-01A-11R-1774-07	Alive	614	5.09575567
TCGA-D5-6530-01A-11R-1723-07	Alive	621	6.71393908
TCGA-D5-6531-01A-11R-1723-07	Alive	540	5.20095548
TCGA-D5-6532-01A-11R-1723-07	Alive	555	5.23188164
TCGA-D5-6533-01A-11R-1723-07	Alive	775	6.69296136
TCGA-D5-6534-01A-21R-1928-07	Alive	1316	4.04063823
TCGA-D5-6535-01A-11R-1723-07	Alive	460	5.85707281
TCGA-D5-6536-01A-11R-1723-07	Alive	543	6.98138233
TCGA-D5-6537-01A-11R-1723-07	Dead	146	5.5664864
TCGA-D5-6538-01A-11R-1723-07	Alive	521	5.86517155
TCGA-D5-6539-01A-11R-1723-07	Alive	380	5.38115183
TCGA-D5-6540-01A-11R-1723-07	Alive	491	6.00985548
TCGA-D5-6541-01A-11R-1723-07	Alive	474	5.09206786
TCGA-D5-6898-01A-11R-1928-07	Alive	229	5.29056816
TCGA-D5-6920-01A-11R-1928-07	Alive	377	4.21027196
TCGA-D5-6922-01A-11R-1928-07	Alive	308	6.29583321
TCGA-D5-6923-01A-11R-A32Z-07	Alive	378	4.59956179
TCGA-D5-6924-01A-11R-1928-07	Alive	435	5.97269036
TCGA-D5-6926-01A-11R-1928-07	Alive	275	6.1835282
TCGA-D5-6927-01A-21R-1928-07	Alive	287	5.08777256
TCGA-D5-6928-01A-11R-1928-07	Alive	354	5.57354722
TCGA-D5-6929-01A-31R-1928-07	Alive	408	5.90187815
TCGA-D5-6930-01A-11R-1928-07	Alive	406	6.4559995
TCGA-D5-6931-01A-11R-1928-07	Alive	365	5.34787113
TCGA-D5-6932-01A-11R-1928-07	Alive	346	6.20006486
TCGA-D5-7000-01A-11R-A32Z-07	Alive	312	5.67481654
TCGA-DC-4745-01A-01R-A32Z-07	Alive	639	6.08799951
TCGA-DC-4749-01A-01R-1736-07	Alive	762	6.14229702
TCGA-DC-5337-01A-01R-1660-07	Alive	792	5.50467441
TCGA-DC-5869-01A-01R-1660-07	Alive	943	5.90413964
TCGA-DC-6154-01A-31R-1928-07	Alive	365	6.36390346
TCGA-DC-6155-01A-11R-1660-07	Alive	425	4.17467806
TCGA-DC-6156-01A-11R-1660-07	Alive	943	5.52529528
TCGA-DC-6157-01A-11R-1660-07	Alive	1581	6.57646016
TCGA-DC-6158-01A-11R-1660-07	Dead	334	5.52677934

TCGA-DC-6160-01A-11R-1660-07	Alive	1339	5.35826614
TCGA-DC-6681-01A-11R-A32Z-07	Alive	790	5.59445599
TCGA-DC-6682-01A-11R-1830-07	Alive	762	6.11503949
TCGA-DC-6683-01A-11R-1830-07	Alive	762	6.6827334
TCGA-DM-A0X9-01A-11R-A155-07	Alive	3641	5.40971678
TCGA-DM-A0XD-01A-12R-A155-07	Dead	743	4.30961277
TCGA-DM-A0XF-01A-11R-A155-07	Dead	1162	5.84518894
TCGA-DM-A1D0-01A-11R-A155-07	Alive	3974	4.83489321
TCGA-DM-A1D4-01A-21R-A155-07	Dead	2821	4.26785786
TCGA-DM-A1D6-01A-21R-A155-07	Dead	1518	7.09176858
TCGA-DM-A1D7-01A-11R-A155-07	Dead	405	5.98390794
TCGA-DM-A1D8-01A-11R-A155-07	Dead	383	5.5405233
TCGA-DM-A1D9-01A-11R-A155-07	Alive	4270	6.26230912
TCGA-DM-A1DA-01A-11R-A155-07	Dead	228	6.39342135
TCGA-DM-A1DB-01A-11R-A155-07	Dead	1348	5.80944531
TCGA-DM-A1HA-01A-11R-A155-07	Alive	4000	0.63291961
TCGA-DM-A1HB-01A-21R-A180-07	Alive	4126	6.113663
TCGA-DM-A280-01A-12R-A16W-07	Dead	236	3.95305101
TCGA-DM-A282-01A-12R-A16W-07	Alive	4233	6.40021684
TCGA-DM-A285-01A-11R-A16W-07	Dead	179	4.25805152
TCGA-DM-A288-01A-11R-A16W-07	Dead	427	5.67145756
TCGA-DM-A28A-01A-21R-A32Y-07	Dead	805	6.72513036
TCGA-DM-A28C-01A-11R-A32Y-07	Dead	2475	4.46609196
TCGA-DM-A28E-01A-11R-A32Y-07	Alive	3648	5.9222597
TCGA-DM-A28F-01A-11R-A32Y-07	Dead	1094	6.31757542
TCGA-DM-A28G-01A-11R-A16W-07	Dead	1849	6.08151859
TCGA-DM-A28H-01A-11R-A16W-07	Alive	3561	4.65187831
TCGA-DM-A28K-01A-21R-A32Y-07	Alive	2988	6.96174023
TCGA-DM-A28M-01A-12R-A16W-07	Alive	2895	4.82744306
TCGA-DT-5265-01A-21R-1830-07	Alive	384	6.24254344
TCGA-DY-A0XA-01A-11R-A155-07	Alive	3846	6.52095177
TCGA-DY-A1DC-01A-31R-A155-07	Dead	1258	6.08174226
TCGA-DY-A1DD-01A-21R-A155-07	Dead	1741	5.07916474
TCGA-DY-A1DE-01A-11R-A155-07	Alive	3932	7.10750375
TCGA-DY-A1DF-01A-11R-A155-07	Dead	734	4.13920752
TCGA-DY-A1DG-01A-11R-A32Y-07	Dead	1566	5.88881873
TCGA-DY-A1H8-01A-21R-A155-07	Dead	992	6.27868919
TCGA-EF-5830-01A-01R-1660-07	Alive	106	5.86713292
TCGA-EF-5831-01A-01R-1660-07	Alive	127	6.2802734
TCGA-EI-6506-01A-11R-1736-07	Alive	625	5.73663464
TCGA-EI-6507-01A-11R-1736-07	Alive	607	7.32506975
TCGA-EI-6508-01A-11R-1736-07	Alive	636	6.05165487
TCGA-EI-6509-01A-11R-1736-07	Alive	517	6.36134201

TCGA-EI-6510-01A-11R-1736-07	Alive	556	6.44816257
TCGA-EI-6511-01A-11R-1736-07	Alive	482	5.72554486
TCGA-EI-6512-01A-11R-1736-07	Alive	538	6.60759811
TCGA-EI-6513-01A-21R-1736-07	Alive	497	5.39077429
TCGA-EI-6514-01A-11R-1736-07	Alive	496	5.55445992
TCGA-EI-6881-01A-11R-A32Z-07	Alive	499	5.44377571
TCGA-EI-6882-01A-11R-1928-07	Alive	262	7.57500064
TCGA-EI-6883-01A-31R-1928-07	Alive	350	6.80776449
TCGA-EI-6884-01A-11R-1928-07	Alive	328	6.10306116
TCGA-EI-6885-01A-11R-1928-07	Alive	415	7.00638219
TCGA-EI-6917-01A-11R-1928-07	Alive	531	5.02336182
TCGA-EI-7002-01A-11R-1928-07	Alive	364	6.46193285
TCGA-EI-7004-01A-11R-1928-07	Alive	257	6.85645416
TCGA-F4-6459-01A-11R-1774-07	Dead	262	5.7430652
TCGA-F4-6460-01A-11R-1774-07	Dead	972	6.43210422
TCGA-F4-6461-01A-11R-1774-07	Dead	338	6.42679812
TCGA-F4-6463-01A-11R-1723-07	Alive	1087	6.27295631
TCGA-F4-6569-01A-11R-1774-07	Alive	1087	3.49573353
TCGA-F4-6570-01A-11R-1774-07	Dead	188	5.21757584
TCGA-F4-6703-01A-11R-1839-07	Alive	1456	3.39160277
TCGA-F4-6704-01A-11R-1839-07	Alive	47	7.44421069
TCGA-F4-6805-01A-11R-1839-07	Alive	1047	5.52344591
TCGA-F4-6806-01A-11R-1839-07	Alive	1260	6.13539122
TCGA-F4-6807-01A-11R-1839-07	Alive	1309	6.00575253
TCGA-F4-6808-01A-11R-1839-07	Alive	1024	5.79223848
TCGA-F4-6809-01A-11R-1839-07	Dead	403	6.64313322
TCGA-F4-6854-01A-11R-1928-07	Alive	16	6.25662952
TCGA-F4-6855-01A-11R-1928-07	Alive	1442	6.50981617
TCGA-F4-6856-01A-11R-1928-07	Alive	1074	6.71906568
TCGA-F5-6464-01A-11R-1736-07	Dead	303	5.03037139
TCGA-F5-6465-01A-11R-1736-07	Alive	1506	4.90432031
TCGA-F5-6571-01A-12R-1830-07	Alive	1288	5.17759494
TCGA-F5-6702-01A-11R-1830-07	Alive	452	5.27160869
TCGA-F5-6811-01A-11R-1830-07	Alive	979	6.08414507
TCGA-F5-6812-01A-11R-1830-07	Alive	1110	6.13783342
TCGA-F5-6813-01A-11R-1830-07	Dead	598	6.4819673
TCGA-F5-6814-01A-31R-1928-07	Alive	1131	5.92667078
TCGA-F5-6861-01A-11R-1928-07	Alive	1160	6.68185402
TCGA-F5-6863-01A-11R-1928-07	Dead	361	6.79240639
TCGA-F5-6864-01A-11R-1928-07	Alive	379	4.98496752
TCGA-G4-6293-01A-11R-1723-07	Alive	4051	3.83216089
TCGA-G4-6294-01A-11R-1774-07	Dead	858	5.72640892
TCGA-G4-6295-01A-11R-1723-07	Alive	254	6.95136515

TCGA-G4-6297-01A-11R-1723-07	Alive	2506	6.05945911
TCGA-G4-6298-01A-11R-1723-07	Dead	715	7.08194673
TCGA-G4-6299-01A-11R-1774-07	Alive	2268	6.34568733
TCGA-G4-6302-01A-11R-1723-07	Dead	2047	4.00565149
TCGA-G4-6303-01A-11R-1774-07	Dead	2003	6.26116032
TCGA-G4-6304-01A-11R-1928-07	Alive	1631	4.59743583
TCGA-G4-6306-01A-11R-1774-07	Alive	1359	4.72764822
TCGA-G4-6307-01A-11R-1723-07	Alive	1674	6.4683145
TCGA-G4-6309-01A-21R-1839-07	Alive	2600	4.55257381
TCGA-G4-6310-01A-11R-1723-07	Alive	1935	5.92344397
TCGA-G4-6311-01A-11R-1723-07	Alive	1199	5.20042981
TCGA-G4-6314-01A-11R-1723-07	Alive	1093	7.02126095
TCGA-G4-6315-01A-11R-1723-07	Alive	1883	6.41536151
TCGA-G4-6317-01A-11R-1723-07	Alive	1095	5.94489592
TCGA-G4-6317-02A-11R-2066-07	Alive	1095	6.45354556
TCGA-G4-6320-01A-11R-1723-07	Alive	804	6.88446971
TCGA-G4-6321-01A-11R-1723-07	Alive	672	4.29592869
TCGA-G4-6322-01A-11R-1723-07	Alive	792	7.76499894
TCGA-G4-6323-01A-11R-1723-07	Alive	419	5.38454375
TCGA-G4-6586-01A-11R-1774-07	Alive	1089	4.87440846
TCGA-G4-6588-01A-11R-1774-07	Alive	796	7.16726358
TCGA-G4-6625-01A-21R-1774-07	Alive	2792	6.26301374
TCGA-G4-6626-01A-11R-1774-07	Dead	1422	7.50552894
TCGA-G4-6627-01A-11R-1774-07	Alive	2275	3.88835361
TCGA-G4-6628-01A-11R-1839-07	Alive	2424	7.27148264
TCGA-G5-6233-01A-11R-1736-07	Dead	556	6.24395464
TCGA-G5-6235-01A-11R-1736-07	Alive	1696	5.66544678
TCGA-G5-6572-01A-11R-1830-07	Dead	1432	6.57765239
TCGA-G5-6572-02A-12R-1830-07	Dead	1432	5.50444878
TCGA-G5-6641-01A-11R-A32Z-07	Alive	804	7.14195895
TCGA-NH-A50T-01A-11R-A28H-07	Alive	553	4.44250565
TCGA-NH-A50U-01A-33R-A37K-07	Dead	334	4.5971557
TCGA-NH-A50V-01A-11R-A28H-07	Alive	588	5.48767928
TCGA-NH-A5IV-01A-42R-A37K-07	Alive	588	5.83549464
TCGA-NH-A6GA-01A-11R-A37K-07	Dead	302	5.4532871
TCGA-NH-A6GB-01A-11R-A37K-07	Alive	476	4.41565727
TCGA-NH-A6GC-01A-12R-A41B-07	Alive	389	6.80253446
TCGA-NH-A8F7-01A-11R-A41B-07	Alive	543	4.41624525
TCGA-NH-A8F7-06A-31R-A41B-07	Alive	543	5.28325508
TCGA-NH-A8F8-01A-72R-A41B-07	Dead	511	5.3106455
TCGA-QG-A5YV-01A-11R-A28H-07	Alive	1301	6.36392623
TCGA-QG-A5YW-01A-11R-A28H-07	Alive	896	5.17511337
TCGA-QG-A5YX-01A-11R-A28H-07	Alive	1003	3.8779209

TCGA-QG-A5Z1-01A-11R-A28H-07	Dead	256	5.71550629
TCGA-QG-A5Z2-01A-11R-A28H-07	Alive	952	6.23518327
TCGA-QL-A97D-01A-12R-A41B-07	Alive	666	5.31594656
TCGA-RU-A8FL-01A-11R-A37K-07	Alive	1177	5.24573903
TCGA-SS-A7HO-01A-21R-A37K-07	Alive	1829	6.70578581
TCGA-T9-A92H-01A-11R-A37K-07	Alive	362	6.21932924
TCGA-WS-AB45-01A-11R-A41B-07	Alive	2130	5.53303688j
